# Validation and Recalibration of PCE, China-PAR, and PREVENT Models for Estimating ASCVD Risk in China

**DOI:** 10.1016/j.jacasi.2025.12.015

**Published:** 2026-02-20

**Authors:** Haibin Li, Shuohua Chen, Ruolin Zhang, Xue Tian, Shouling Wu, Anxin Wang

**Affiliations:** a Department of Cardiology, Beijing Chaoyang Hospital, Capital Medical University, Beijing, China; b Medical Research Center, Beijing Chaoyang Hospital, Capital Medical University, Beijing, China; c Department of Cardiology, Kailuan Hospital, North China University of Science and Technology, Tangshan, China; d Harvard T.H. Chan School of Public Health, Harvard University, Boston, Massachusetts, USA; e Department of Epidemiology, Beijing Neurosurgical Institute, Beijing Tiantan Hospital, Capital Medical University, Beijing, China; f China National Clinical Research Center for Neurological Diseases, Beijing Tiantan Hospital, Capital Medical University, Beijing, China; g Department of Clinical Epidemiology and Clinical Trial, Capital Medical University, Beijing, China

**Keywords:** atherosclerotic cardiovascular disease, population-based cohort study, recalibration, risk score, validation

## Abstract

**Background:**

The Pooled Cohort Equations (PCE) and Prediction for Atherosclerotic Cardiovascular Disease (ASCVD) Risk in China (China-PAR) models tend to overestimate risk, whereas the Predicting Risk of Cardiovascular Disease Events (PREVENT) equations may underestimate risk in many contemporary cohorts.

**Objectives:**

This study aimed to validate and determine if recalibration of these risk scores using contemporary population-level data improves risk stratification for primary prevention in China.

**Methods:**

These risk scores were first validated and then recalibrated in the Kailuan study. Participants aged 40 to 79 years without ASCVD at baseline were included. Original and recalibrated models were assessed for discrimination and calibration.

**Results:**

Of 79,497 participants, 4,425 ASCVD events occurred over a median follow-up of 10 years (Q1-Q3: 10-10 years). All 3 original models showed good discrimination in women (Harrell’s C-index: PCE 0.735 [95% CI: 0.712-0.757], China-PAR 0.738 [95% CI: 0.715-0.760], PREVENT 0.737 [95% CI: 0.713-0.759]) and moderate discrimination in men (PCE 0.675 [95% CI: 0.667-0.683], China-PAR 0.685 [95% CI: 0.677-0.693], and PREVENT 0.685 [95% CI: 0.677-0.693]). Original models showed differential mean calibration in 10-year ASCVD risk estimation: PCE overestimated by 34.9% (men [95% CI: 32.8%-36.9%]) and 15.1% (women [95% CI: 6.8%-22.7%]); China-PAR by 17.7% (men [95% CI: 15.1%-20.2%]) and 48.2% (women [95% CI: 43.1%-52.8%]); PREVENT underestimated by 29.4% (men [95% CI: 25.4%-33.5%]) and 2.7% (women [95% CI: −6.5% to 12.8%]). After recalibrating to the local population, observed vs predicted risks exhibited improved alignment for the recalibrated models.

**Conclusions:**

In this Chinese cohort, the original PCE and China-PAR overestimated 10-year ASCVD risk, whereas PREVENT underestimated it. Recalibration mitigated such misestimation in the local population, potentially enhancing risk stratification in primary cardiovascular prevention in China.

Atherosclerotic cardiovascular disease (ASCVD), a major global public health issue, is the leading cause of premature morbidity and mortality worldwide,^1^ particularly in China.^2^ In recent years, risk factors associated with ASCVD, such as obesity, hypertension, and physical inactivity, have been increasing.^3^, ^4^, ^5^ If the levels of risk factors remain unchanged, the incidence of cardiovascular events per year is projected to increase by over 50% between 2010 and 2030, primarily caused by population aging and growth.^6^

Population-based risk assessments are crucial for the primary prevention and management of ASCVD. Preventive interventions, such as statin therapy, can be effectively guided by precise risk prediction models grounded in established clinical guidelines.^7^ The Pooled Cohort Equations (PCE), released in 2013 by the American College of Cardiology and the American Heart Association, constitutes one of the most widely used risk prediction models for estimating 10-year ASCVD risks.^8^ Originally developed using data from 5 nationwide cohorts, the PCE has been well-recalibrated around decision thresholds for the U.S. population.^9^^,^^10^ However, the performance of the PCE varies significantly among different racial and ethnic groups.^11^, ^12^, ^13^, ^14^, ^15^ To address this challenge, the American Heart Association developed new ASCVD risk prediction equations in 2023, known as the PREVENT (Predicting Risk of Cardiovascular Disease Events) equations.^16^ The limited applicability of Western-developed risk prediction models to the Chinese population may be caused by differences in the distribution of ASCVD forms between these populations. Therefore, to assessment and management of cardiovascular risk in China, the China-PAR (Prediction for ASCVD risk in China) model was developed in 2016.^17^ The China-PAR model was derived from two large prospective cohort studies conducted in 1998-2001. However, the China-PAR model demonstrated an underestimation of risk by 20% in men and 40% in women within a contemporary Chinese cohort.^18^ In China, the high prevalence of hypertension contributes to a stroke-dominant ASCVD profile,^19^ whereas in Western countries, coronary heart disease predominates in ASCVD cases,^20^ affecting the relative risks associated with ASCVD risk factors. Additionally, China’s rapidly changing cardiovascular health landscape, driven by economic development, an aging population, and evolving lifestyles, highlights the inadequacy of directly applying Western models to the Chinese context. Therefore, evaluating and recalibrating the PCE, China-PAR, and PREVENT is crucial for the contemporary Chinese population.

Recalibrating a risk prediction tool using population-based cohort data ensures its accuracy and applicability across diverse individuals within that population. By using comprehensive and representative data, the recalibrated tool can provide more precise and relevant risk predictions for the entire target group. In Austria, the recalibration of the PCE using contemporary cohort data significantly improved their calibration, particularly for high-risk individuals, with discrimination c-statistics ranging from 0.73 to 0.79.^21^ A study on the Korean population demonstrated that integrating multipolygenic risk scores with recalibrated PCE provided better ASCVD risk stratification, although the predictive improvement was marginal.^22^ However, the evidence for PCE recalibration in Chinese populations is limited, and the calibration effects are divergent.^11^^,^^18^^,^^23^ In addition, PREVENT equations have not been sufficiently validated externally so far in the Chinese population.

The purpose of this study was to validate and recalibrate the PCE, China-PAR, and PREVENT using a contemporary population-based cohort. We compared the original model with the recalibrated model to evaluate the agreement between observed and predicted ASCVD events rate, thereby providing health care providers with a more precise tool. By improving risk assessment, the recalibration facilitates better identification of high-risk patients who may benefit from preventive interventions, leading to more personalized and effective prevention strategies, potentially reducing the incidence of cardiovascular events, and improving overall cardiovascular health outcomes in China.

## Methods

### Study populations

**Figure 1:**
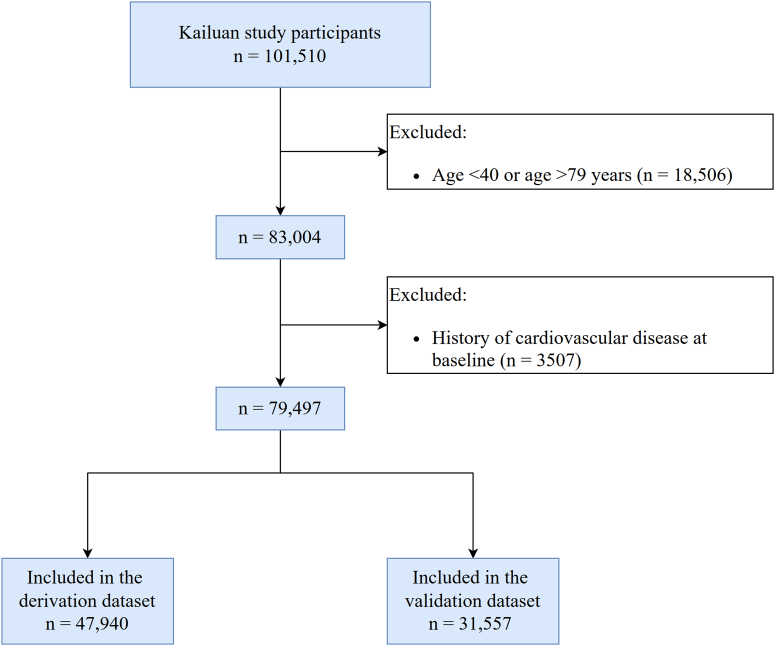
Selection and Characteristics of the Study Population Graphical overview of cohort selection. We first validated the Pooled Cohort Equations (PCEs), Prediction of atherosclerotic cardiovascular disease risk in China (China-PAR), and Predicting Risk of Cardiovascular Disease EVENTs (PREVENT) model in the whole cohort. To avoid overfitting, we randomly split our cohort into the derivation set (60%) and the validation set (40%). Original PCE, China-PAR, and PREVENT model were recalibrated in the derivation set and validated in the validation set.

The recalibration cohort was derived from the Kailuan study, an ongoing prospective cohort study. A full description of the design and objectives of the Kailuan study has been published.^24^^,^^25^ Briefly, at baseline (June 2006 and October 2007), a total of 101,510 men and women, aged 18 to 98 years, from the Kailuan community in China were recruited. Participants have performed questionnaires and health assessments every 2 years since the baseline survey. For the current study, a total of 79,497 participants were included, after the exclusion of those younger than 40 years or older than 80 years at baseline (n = 18,506) and with a history of cardiovascular diseases (n = 3,507) (Figure 1). All participants provided written informed consent, and the protocol was approved by the ethics committee of the Kailuan General Hospital, and the Beijing Tiantan Hospital, Capital Medical University.

### Measurements

Data collection in the Kailuan study included questionnaires, physical examinations, and blood sample. The following variables were extracted from the Kailuan study: demographic characteristics (age and sex), cardiovascular risk factors (eg, smoking status, diabetes, and hypertension, and family history of ASCVD), medication use (eg, antihypertensive and lipid-lowering medication), and laboratory values (eg, fasting blood glucose [FBG], total cholesterol, high-density lipoprotein cholesterol, low-density lipoprotein cholesterol, triglycerides, creatinine, and high-sensitivity C-reactive protein [hsCRP]). Smoking status was stratified into 3 levels: current smoker, former smoker, and never. Family history of ASCVD was defined as at least 1 of their parents diagnosed with myocardial infarction or stroke. Trained staff measured participants’ height, weight, waist circumference, systolic blood pressure, and diastolic blood pressure. Body mass index (kg/m^2^) was calculated as weight (kilograms) divided by height squared (meters). Diabetes mellitus was defined as a self-reported physician-diagnosis history, currently treated with insulin or oral hypoglycemic agents, or a FBG concentration ≥7.0 mmol/L. We calculated the estimated glomerular filtration rate using the Chronic Kidney Disease Epidemiology Collaboration (CKD-EPI 2009) creatinine equation.^26^ The triglyceride glucose (TyG) index was calculated as Ln (triglycerides [mg/dL] × FBG [mg/dL]/2), which is an easily accessible surrogate marker of insulin resistance.^27^

### Outcomes

The incident ASCVD events were defined as myocardial infarction and ischemic stroke. As described previously,^24^^,^^25^ participants enrolled in the Kailuan study received biannual questionnaires and health examinations to gather information on the occurrence of ASCVD during follow-up. Additional information was collected from the Municipal Social Insurance Institution and Hospital Discharge Register. Mortality information was gathered from provincial vital statistics offices. To identify potential ASCVD events, we reviewed hospital discharge lists from 11 local hospitals and asked for a history of ASCVD using questionnaires in the biennial interview. For all suspected ASCVD events, an endpoint committee of 3 physicians adjudicated all clinical events independently.

### Statistical analysis

Baseline characteristics are presented as the mean ± SD, median (Q1-Q3), or n (%). Extreme values (eg, below percentile 1 and above percentile 99) were Winsorized to limit their influence. The missingness percentages of covariates ranged from 1.2% to 3.6% (Supplemental Table 1). We hypothesized that the missingness in covariates was missing at random if not completely at random. We performed multiple imputation by chained equations to address the missing data.^28^ We produced 5 imputed data sets, and the estimates from each imputed data set were combined into one overall estimate with the use of Rubin’s rule. The details in the settings of multiple imputation are provided in the Supplemental Methods.

Performance metrics were evaluated at 10 years. Participants were censored at the time the first of the following events occurred: 1) a first ASCVD event; 2) death; and 3) 10 years of follow-up. For each participant, we first calculated the predicted 10-year ASCVD risk using the original PCE (Caucasian),^29^ China-PAR,^17^ and PREVENT (base)^16^ equation (Supplemental Table 2) for men and women. We then evaluated the performance of the original PCE, China-PAR, and PREVENT equation in terms of discrimination, calibration, and overall performance. Harrell’s C-index assesses the model’s discriminatory ability to separate individuals with ASCVD from those without ASCVD, ranging from 0.5 (indicating no discrimination) to 1 (indicating perfect discrimination).^30^ Calibration refers to how closely the predicted 10-year ASCVD risk agrees with the observed 10-year ASCVD risk.^31^ Calibration was evaluated in 3 levels: 1) mean calibration; 2) weak calibration; and 3) moderate calibration.^32^ Mean calibration (or “calibration-in-the-large”) is estimated as the ratio of predicted to observed event rates. The 10-year observed ASCVD risk was estimated using the Kaplan-Meier method. To assess weak calibration at a fixed time point, we fitted a “secondary” Cox model with the “Individual Score” as the only covariate and reported the calibration slope. Moderate assessment of calibration (or “calibration-in-the-small”) focuses whether the observed outcome rate equals the predicted risk among participants with the same predicted risk. Calibration metrics, such as Integrated Calibration Index (ICI), E50, and E90, were calculated from the smooth calibration curve. The ICI is the mean absolute difference between smoothed observed proportions and predicted probabilities. The E50 and E90 statistics denote the median and the 90th percentile absolute difference between observed and predicted probabilities of the outcome. Calibration performance was also assessed graphically by categorizing participants into quintiles of predicted 10-year ASCVD risk and plotting mean 10-year predicted risk against observed 10-year risk. For overall performance, we reported the Brier and Index of Prediction Accuracy (IPA) score.^32^ The Brier score encompasses both discrimination and calibration. The IPA score is calculated based on the model’s predicted risks, which reflects an *R*^*2*^-type assessment. We used nonparametric bootstrapping with 200 replications to calculate percentile-based 95% CIs for the ICI, E50, E90, Brier, and IPA score.

Recalibration is typically necessary when evaluating a prediction model in a new population with a different underlying event rate.^31^ To avoid overfitting, we randomly split our Kailuan cohort into the derivation set (60%) and the validation set (40%). In the derivation set, the original PCE and China-PAR model was recalibrated via replacing the baseline hazard and mean risk factor levels (Method 1)^12^ and performing logistic recalibration (Method 2)^33^ separately by sex. The PCE and China-PAR model was obtained from the sex-specific Cox proportional hazards regression. The risk equation is as follows: $1-S_{0}{\left(t\right)}^{\exp\left(\sum_{i=1}^{p}\left(\beta_{i}\cdot x_{i}-\beta_{i}\cdot \bar{x_{i}}\right)\right)}$, where ${\;S}_{0}\left(t\right)$ is the baseline survival function freedom from ASCVD event at time *t* (eg, 10 years) for the developed population with average risk factors, $\beta_{i}$ is the regression coefficients, $x_{i}$ is the risk predictor values for a given individual, $\bar{x_{i}}$ is the mean or proportion for each risk predictor, and $\beta_{i}\cdot x_{i}$ is the linear predictor. The PCE and China-PAR model was recalibrated by replacing $S_{0}\left(t\right)$ with the average freedom from ASCVD at 10 years in the derivation cohort (calculating by fitting the linear predictor as an offset term in Cox model), and replacing $\bar{x_{i}}$ for each predictor with the mean or proportion for the same risk predictor in the derivation cohort.^12^ The logistic recalibration was performed by fitting a Cox proportional hazards model. The linear predictor was the sole parameter included, and the baseline survival at 10 years was estimated with the linear predictor set to the mean level. Subsequently, the PCE and China-PAR model was updated by substituting the current baseline survival value with the updated one, and multiplying the linear predictor by the model’s coefficient. The risk equation for PREVENT model is as follows: $\frac{e^{\log\;odds}}{1+e^{\log\;odds}}$, $where\;\log\;odds=\sum_{i=1}^{p}\left({\beta_{0}+\beta }_{i}\cdot x_{i}\right)$. The original PREVENT model was recalibrated firstly by fitting a logistic regression model with the linear predictor as offset term in the derivation set (Method 1: recalibration in the large).^33^ We then adjusted the intercept and all predictor regression coefficients of PREVENT base equations by 1 overall adjustment factor (eg, calibration slope) by fitting a new logistic regression model in the derivation set including the linear predictor as the only predictor (Method 2: Logistic recalibration).^33^^,^^34^ The original regression coefficients $\left(\beta_{i}\right)$ were used and not re-estimated in the stage of recalibration. The recalibrated risk equations were summarized in Supplemental Tables 3 and 4.

In the validation set, we evaluated the calibration and discrimination of the recalibrated equations and compared them with the 10-year ASCVD risk estimates of the original equations. The decision curve analysis was conducted to assess the clinical performance of recalibrated and original risk scores.

Last, we updated the original model by fitting Cox regression models, including 2 new biomarkers (TyG index and hsCRP) in addition to the linear predictor of PCE, China-PAR, and PREVENT, respectively. To verify proportional hazards assumption, scaled Schoenfeld residuals were plotted over time and visually inspected for nonproportionality (Supplemental Figure 1). Minor deviations observed in the Schoenfeld plots were deemed acceptable when their magnitude was considered negligible and unlikely to impact the estimates, particularly given the substantially large sample size. We also plotted the survival curves and visually inspected for consistent, noncrossing separation between the groups (Supplemental Figure 2). We evaluated and compared incremental predictive values of the revised model with the original PCE, China-PAR, and PREVENT model using Harrell’s C-index, calibration curves, and decision curves in the whole Kailuan cohort.

We adhered to the Transparent Reporting of a Multivariable Prediction Model for Individual Prognosis or Diagnosis (TRIPOD) statement to assess the model performance.^35^ Statistical significance was defined as 2-sided *P <* 0.05. All analyses were performed using SAS version 9.4 and R version 4.5.1.

## Results

### Baseline characteristics

Among 79,497 participants without prior ASCVD eligible for the main analysis, the mean age was 54.8 ± 9.0 years, and 63,845 (80.3%) were men (Table 1). Overall, men were older, had a higher prevalence of smoking, and had a lower proportion of individuals taking antihypertensive and lipid-lowering medication compared with women (Supplemental Table 5). Based on the PCE, China-PAR, and PREVENT (base) equation, the median predicted 10-year ASCVD risk was 5.5%, 5.4%, and 3.2%, respectively. The distribution of 10-year ASCVD risk by sex was shown in the Supplemental Figure 3. The baseline characteristics of participants in the validation set (n = 47,940) were similar to those of the derivation set (n = 31,557) (Supplemental Table 6). During the 10-year follow-up (Q1-Q3: 10-10 years), 437 (2.8%) of 15,652 women and 3,988 (6.2%) of 63,845 men had an incident ASCVD events in whole cohort. In the derivation set, 2,388 of (6.2%) 38,491 men and 266 (2.8%) of 9,449 women had ASCVD events. In the validation set, 1,600 (6.3%) of 25,354 men and 171 (2.8%) of 6,203 women had ASCVD events.

**Table 1:** Baseline Characteristics in the Derivation and Validation Set, by Sex

	Overall (N = 79,497)	Derivation Set		Validation Set	
		Women (n = 9,449)	Men (n = 38,491)	Women (n = 6,203)	Men (n = 25,354)
Age, y	54.8 ± 9.0	52.7 ± 8.2	55.3 ± 9.0	52.8 ± 8.3	55.3 ± 9.1
Current smoker	26,033 (34.0)	188 (2.1)	15,508 (41.8)	126 (2.1)	10,211 (41.8)
Diabetes mellitus	8,128 (10.2)	929 (9.8)	4,010 (10.4)	550 (8.9)	2,639 (10.4)
Antihypertensive medication	8,935 (11.2)	1,232 (13.0)	4,158 (10.8)	804 (13.0)	2,741 (10.8)
Lipid-lowering medication	654 (0.8)	122 (1.3)	293 (0.8)	68 (1.1)	171 (0.7)
Family history of ASCVD	4,690 (5.9)	722 (7.6)	2,122 (5.5)	477 (7.7)	1,369 (5.4)
Blood pressure, mm Hg					
Systolic	132.7 ± 20.5	127.9 ± 20.6	133.9 ± 20.3	128.2 ± 20.6	133.8 ± 20.2
Diastolic	84.4 ± 11.5	80.9 ± 10.9	85.2 ± 11.5	81.0 ± 10.7	85.2 ± 11.6
BMI, kg/m^2^	25.1 ± 3.3	25.1 ± 3.5	25.1 ± 3.2	25.0 ± 3.5	25.1 ± 3.2
Waist circumference, cm	87.7 ± 9.3	84.8 ± 9.7	88.4 ± 9.1	84.6 ± 9.6	88.4 ± 9.0
Cholesterol, mmol/L					
Total	5.0 ± 1.1	5.1 ± 1.1	5.0 ± 1.1	5.1 ± 1.0	5.0 ± 1.1
HDL	1.6 ± 0.4	1.6 ± 0.4	1.6 ± 0.4	1.6 ± 0.4	1.6 ± 0.4
LDL	2.3 ± 0.9	2.2 ± 0.9	2.4 ± 0.9	2.2 ± 0.9	2.4 ± 0.9
Triglycerides, mmol/L	1.3 (0.9-2.0)	1.3 (0.9-1.9)	1.3 (0.9-2.0)	1.3 (0.9-1.9)	1.3 (0.9-2.0)
FBG, mmol/L	5.5 ± 1.6	5.4 ±1.7	5.6 ± 1.6	5.3 ± 1.5	5.6 ± 1.6
hsCRP, mg/L	0.8 (0.3-2.3)	0.9 (0.3-2.7)	0.8 (0.3-2.2)	1.0 (0.3-2.8)	0.8 (0.3-2.2)
eGFR, mL/min/1.73 m^2^	79.5 (67.0-93.6)	76.0 (64.1-89.8)	80.6 (67.8-94.5)	75.8 (64.2-89.3)	80.5 (67.8-94.4)
TyG index	8.7 (0.7)	8.6 (0.7)	8.7 (0.7)	8.6 (0.6)	8.7 (0.7)
10-y incident ASCVD	4,425 (5.6)	266 (2.8)	2,388 (6.2)	171 (2.8)	1,600 (6.3)
PCE 10-y ASCVD risk, %	5.5 (2.3-11.4)	1.4 (0.7-3.2)	6.8 (3.5-13.0)	1.4 (0.7-3.2)	6.8 (3.5-13.0)
China-PAR 10-y ASCVD risk, %	5.4 (2.9-10.2)	3.6 (1.8-7.4)	5.9 (3.2-10.8)	3.6 (1.8-7.4)	5.9 (3.2-10.8)
PREVENT 10-y ASCVD risk, %	3.2 (1.8-6.1)	1.6 (0.9-3.5)	3.6 (2.1-6.7)	1.6 (0.9-3.4)	3.7 (2.1-6.6)

Values are mean ± SD, n (%), or median (Q1-Q3).

ASCVD = atherosclerotic cardiovascular disease; BMI = body mass index; China-PAR = Prediction of ASCVD risk in China; eGFR = estimated glomerular filtration rate; FBG = fasting blood glucose; HDL = high-density lipoprotein; hsCRP = high-sensitivity C-reactive protein; LDL = low-density lipoprotein; PCE = Pooled Cohort Equation; PREVENT = Predicting Risk of Cardiovascular Disease EVENTs.

### Validation of the original PCE, China-PAR, and PREVENT equation

**Figure 2:**
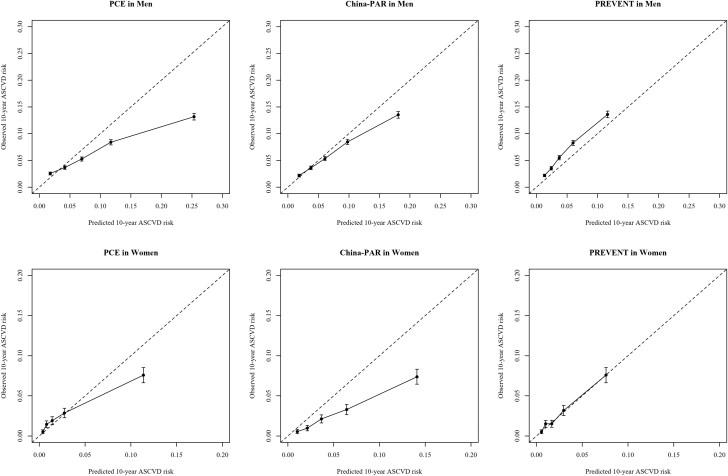
Calibration Plots of Predicted vs Observed Rate in Whole Cohort The observed rate of atherosclerotic cardiovascular disease (ASCVD) events at 10 years was estimated based on the Kaplan-Meier method. Predicted 10-year risk of ASCVD was calculated using original PCE, China-PAR, and PREVENT models. Data points represent mean and error bars are 95% CI. Abbreviations as in Figure 1.

As illustrated in Table 2, in the whole cohort, sex-specific Harrell’s C-index for original PCE were 0.735 (95% CI: 0.712-0.757) in women and 0.675 (95% CI: 0.667-0.683) in men in the whole cohort. These figures were comparable to those observed for the original China-PAR model (0.738; 95% CI: 0.715-0.760 in women and 0.685; 95% CI: 0.677-0.693 in men) and the original PREVENT equation (0.737; 95% CI: 0.713-0.759 in women and 0.685; 95% CI: 0.677-0.693 in men). According to mean calibration, the original PCE overestimated the 10-year risk of ASCVD by 34.9% (95% CI: 32.8%-36.9%) in men and 15.1% (95% CI: 6.8%-22.7%) in women. The original China-PAR model overestimated the 10-year risk of ASCVD by 17.7% (95% CI: 15.1%-20.2%) in men and 48.2% (95% CI: 43.1%-52.8%) in women. Although the original PREVENT equation underestimated the 10-year risk of ASCVD by 29.4% (95% CI: 25.4%-33.5%) in men and 2.7% (95% CI: −6.5% to 12.8%) in women. The calibration curves and other metrics are displayed in Figure 2 and Table 2, respectively.

**Table 2:** Validation of PCE, China-PAR, and PREVENT Equation in Whole Cohort

	Women (n = 15,652)	Men (n = 63,845)
PCE		
Discrimination		
Harrell’s C-index	0.735 (0.712-0.757)	0.675 (0.667-0.683)
Calibration		
Mean calibration	0.849 (0.773-0.932)	0.651 (0.631-0.672)
Weak calibration		
Slope	0.655 (0.584-0.726)	0.653 (0.619-0.687)
Moderate calibration		
ICI	0.012 (0.010-0.014)	0.036 (0.034-0.038)
E50	0.005 (0.003-0.007)	0.015 (0.012-0.017)
E90	0.020 (0.013-0.027)	0.101 (0.096-0.106)
Overall		
Brier	0.024 (0.022-0.027)	0.048 (0.046-0.049)
IPA	0.091 (0.075-0.108)	0.164 (0.156-0.171)
China-PAR Model		
Discrimination		
Harrell’s C-index	0.738 (0.715-0.760)	0.685 (0.677-0.693)
Calibration		
Mean calibration	0.518 (0.472-0.569)	0.823 (0.798-0.849)
Weak calibration		
Slope	0.957 (0.848-1.066)	0.822 (0.781-0.863)
Moderate calibration		
ICI	0.026 (0.023-0.029)	0.014 (0.012-0.016)
E50	0.017 (0.014-0.020)	0.006 (0.003-0.008)
E90	0.060 (0.053-0.067)	0.038 (0.033-0.043)
Overall		
Brier	0.023 (0.021-0.025)	0.051 (0.049-0.053)
IPA	0.142 (0.129-0.156)	0.101 (0.096-0.107)
PREVENT Equation		
Discrimination		
Harrell’s C-index	0.737 (0.713-0.759)	0.685 (0.677-0.693)
Calibration		
Mean calibration	1.027 (0.935-1.128)	1.294 (1.254-1.335)
Weak calibration		
Slope	0.894 (0.795-0.993)	0.827 (0.786-0.867)
Moderate calibration		
ICI	0.003 (0.001-0.005)	0.016 (0.014-0.018)
E50	0.002 (0.000-0.004)	0.017 (0.015-0.019)
E90	0.004 (0.001-0.008)	0.024 (0.019-0.028)
Overall		
Brier	0.025 (0.023-0.028)	0.056 (0.054-0.058)
IPA	0.051 (0.041-0.061)	0.017 (0.012-0.022)

Values in parentheses are 95% CIs.

ASCVD = atherosclerotic cardiovascular disease; China-PAR = Prediction of ASCVD risk in China; E50 = median of absolute difference between observed and predicted probabilities; E90 = 90th percentile of absolute difference between observed and predicted probabilities; ICI = integrated calibration index; IPA = index of prediction accuracy; PCE = Pooled Cohort Equation; PREVENT = Predicting Risk of Cardiovascular Disease EVENTs.

### Recalibration and validation of the PCE, China-PAR, and PREVENT equation

In the validation set, after recalibration to the local Kailuan population in the derivation set, the mean predicted ASCVD rates were mostly closer to the observed ones **(**Central Illustration**)**. The recalibrated mean calibration, Harrell’s C-index, calibration slope, and other metrics in the validation set are displayed in Table 3.

**Central Illustration:**
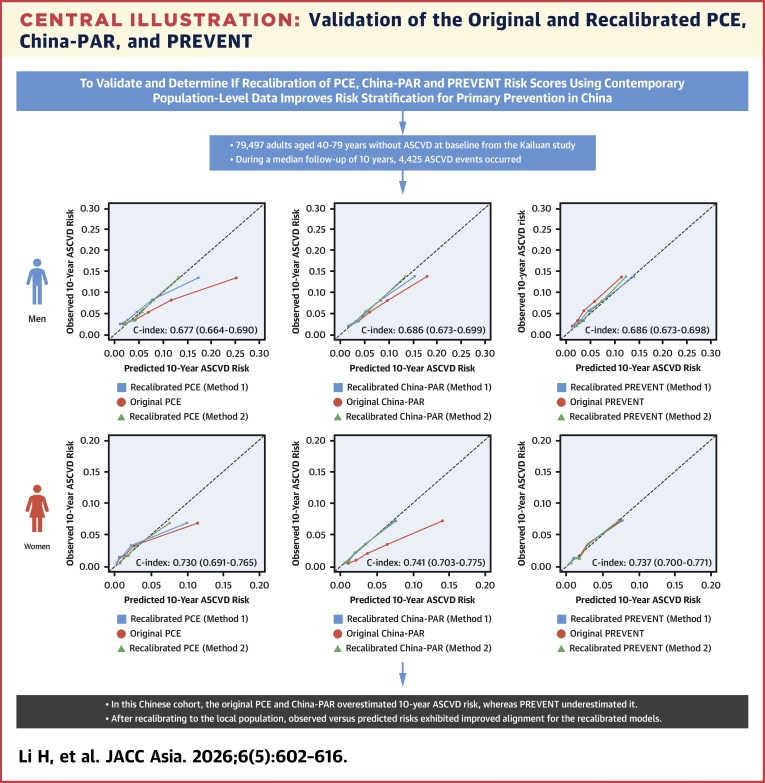
Validation of the Original and Recalibrated PCE, China-PAR, and PREVENT Calibration plots depict the mean observed and predicted 10-year risk of atherosclerotic cardiovascular disease (ASCVD) events across quintiles of predicted risk in the validation set. China-PAR = Prediction of ASCVD risk in China; PCE = Pooled Cohort Equation; PREVENT = Predicting Risk of Cardiovascular Disease EVENTs.

**Table 3:** Performance of the Recalibrated PCE, China-PAR, and PREVENT Equation in Validation Set

	Recalibrated Model (Method 1)		Recalibrated Model (Method 2)	
	Women (n = 6,203)	Men (n = 25,354)	Women (n = 6,203)	Men (n = 25,354)
Recalibrated PCE				
Discrimination				
Harrell’s C-index	0.730 (0.691-0.765)	0.677 (0.664-0.690)	0.730 (0.691-0.765)	0.677 (0.664-0.690)
Calibration				
Mean calibration	0.959 (0.825-1.114)	0.977 (0.930-1.026)	0.962 (0.828-1.117)	0.997 (0.949-1.047)
Weak calibration				
Slope	0.629 (0.517-0.742)	0.655 (0.601-0.708)	0.937 (0.769-1.104)	1.005 (0.923-1.087)
Moderate calibration				
ICI	0.012 (0.008-0.015)	0.015 (0.012-0.018)	0.004 (0.001-0.007)	0.003 (0.001-0.005)
E50	0.007 (0.004-0.010)	0.012 (0.010-0.015)	0.002 (0.000-0.005)	0.002 (0.000-0.004)
E90	0.014 (0.007-0.021)	0.026 (0.018-0.033)	0.006 (0.000-0.012)	0.006 (0.002-0.011)
Overall				
Brier	0.025 (0.021-0.028)	0.053 (0.051-0.056)	0.025 (0.021-0.029)	0.055 (0.052-0.057)
IPA	0.071 (0.050-0.093)	0.073 (0.063-0.083)	0.050 (0.035-0.064)	0.048 (0.040-0.057)
Recalibrated China-PAR Model				
Discrimination				
Harrell’s C-index	0.741 (0.703-0.775)	0.686 (0.673-0.699)	0.741 (0.703-0.775)	0.686 (0.673-0.699)
Calibration				
Mean calibration	0.960 (0.827-1.116)	0.977 (0.930-1.026)	0.969 (0.834-1.126)	1.000 (0.952-1.050)
Weak calibration				
Slope	0.954 (0.781-1.128)	0.826 (0.761-0.891)	0.996 (0.815-1.177)	1.008 (0.928-1.088)
Moderate calibration				
ICI	0.003 (0.000-0.006)	0.007 (0.004-0.009)	0.003 (0.000-0.005)	0.003 (0.001-0.005)
E50	0.002 (−0.000 to 0.004)	0.006 (0.003-0.008)	0.002 (−0.000 to 0.004)	0.002 (0.000-0.004)
E90	0.006 (−0.001 to 0.012)	0.012 (0.005-0.019)	0.005 (−0.001 to 0.011)	0.006 (0.000-0.011)
Overall				
Brier	0.025 (0.021-0.029)	0.054 (0.051-0.056)	0.025 (0.021-0.029)	0.054 (0.052-0.057)
IPA	0.054 (0.040-0.068)	0.067 (0.058-0.076)	0.051 (0.037-0.064)	0.053 (0.045-0.061)
Recalibrated PREVENT Equation				
Discrimination				
Harrell’s C-index	0.737 (0.700-0.771)	0.686 (0.673-0.698)	0.737 (0.700-0.771)	0.686 (0.673-0.698)
Calibration				
Mean calibration	0.994 (0.855-1.154)	1.064 (1.013-1.117)	0.994 (0.856-1.155)	1.063 (1.012-1.116)
Weak calibration				
Slope	0.879 (0.721-1.036)	0.826 (0.763-0.890)	0.977 (0.802-1.153)	1.034 (0.954-1.114)
Moderate calibration				
ICI	0.004 (0.001-0.007)	0.007 (0.004-0.010)	0.003 (0.000-0.006)	0.006 (0.003-0.009)
E50	0.003 (−0.000 to 0.005)	0.007 (0.005-0.010)	0.002 (0.000-0.005)	0.004 (0.001-0.008)
E90	0.007 (0.000-0.013)	0.009 (0.005-0.013)	0.006 (−0.000 to 0.012)	0.014 (0.007-0.022)
Overall				
Brier	0.025 (0.021-0.029)	0.055 (0.052-0.057)	0.025 (0.021-0.029)	0.055 (0.053-0.058)
IPA	0.052 (0.037-0.067)	0.048 (0.039-0.057)	0.047 (0.034-0.060)	0.038 (0.030-0.046)

Values in parentheses are 95% CIs.

Abbreviations as in Table 2.

**Figure 3:**
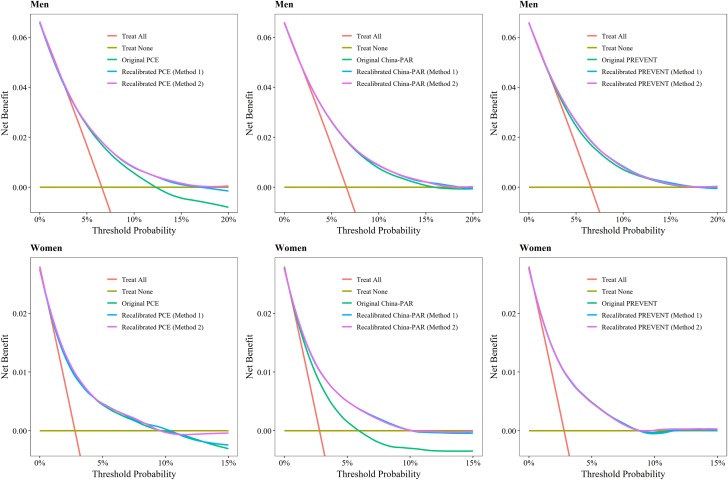
Decision Curves for Original and Recalibrated Models in Validation Set The 10-year treatment probability threshold was calculated using the original and recalibrated the PCE, China-PAR, PREVENT models. Abbreviations as in Figures 1 and 2.

Figure 3 displays the decision curves for 10-year treatment thresholds ranging from 0% to 20% for men and 0% to 15% for women, using the validation set. In men, statin treatment guided by the recalibrated PCE resulted in a greater net benefit compared with treatment based on the original PCE. Similarly, for women, statin treatment informed by the recalibrated China-PAR showed a greater net benefit than that guided by the original China-PAR. No significant differences in benefit were observed between the original and recalibrated PREVENT equation for either men or women.

### Updating of the original PCE, China-PAR, and PREVENT equation

**Figure 4:**
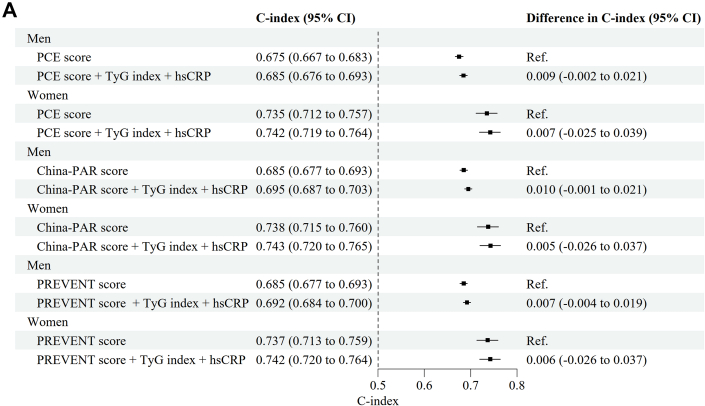

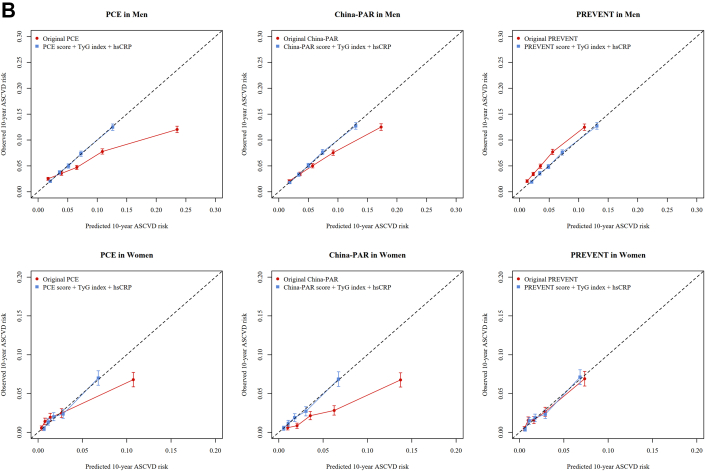

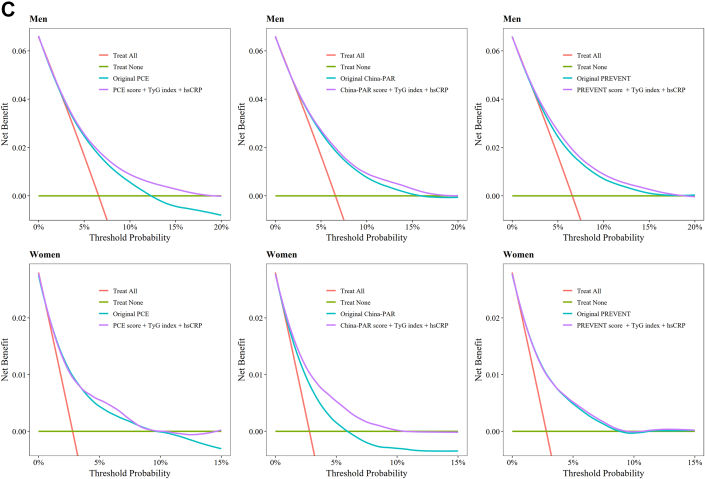
Performance for Original vs Updating Models in Whole Cohort As presented in the forest plots of Harrell’s C-index (A), calibration curves (B), and decision curves (C), the original PCE, China-PAR, and PREVENT models were compared with their updated counterparts. The latter were constructed by integrating 2 key biomarkers—triglyceride-glucose (TyG) index (linked to insulin resistance) and high-sensitivity C-reactive protein (hsCRP) (associated with inflammation)—into the original model. Abbreviations as in Figures 1 and 2.

In the whole cohort, both TyG index and hsCRP were associated with ASCVD in both men and women, after adjusting for PCE, China-PAR, and PREVENT risk scores (Supplemental Table 7). We did not observe that inclusion of TyG index and hsCRP significantly increase the Harrell’s C-index compared with the original PCE, China-PAR, and PREVENT equation, respectively (Figure 4). However, both calibration curves and decision curves demonstrated that the updated model confers significant clinical benefit (Figure 4).

## Discussion

In this large population-based cohort study conducted in China, the PCE, China-PAR, and PREVENT models demonstrated good discrimination in predicting 10-year ASCVD risk among women, while showing only modest discrimination in men. Our findings for the original PCE, China-PAR, and PREVENT models diverged. Namely, the original PCE and China-PAR models substantially overestimated the 10-year ASCVD risk in the highest-risk group for both sexes, while the PREVENT model underestimated the risk in men but showed accurate calibration for women in this Chinese population. Recalibrating the PCE, China-PAR, and PREVENT models improved accuracy so that predicted risks were mostly closer to observed risks.

The original cohort utilized to derive the PCE excluded Asian populations resulted in only moderate accuracy for this population. In a primary care setting in Asia, the PCE was found to overestimate cardiovascular risk compared with the Framingham General Cardiovascular Disease risk score.^36^ Furthermore, the Multi-Ethnic Study of Atherosclerosis demonstrated that the PCE overestimated ASCVD risk across different ethnic groups, with the highest overestimation observed among Chinese participants.^14^ Consistent with previous research, our study demonstrates that the direct application of the PCE overestimates ASCVD risk, especially among men. The discrepancy between predicted and observed risk was most significant in the highest risk group. Notably, overestimation in high-risk populations can lead to unnecessary interventions, such as the excessive use of statin therapy, which may not be justified by the actual risk levels.

Research on the recalibration of PCE in the Chinese population was limited, and most studies indicated that recalibration does not enhance predictive ability.^11^^,^^18^^,^^23^ The study involving 11,169 rural Northern Chinese adults in the “stroke belt” found that the original PCE underestimated risk by 76.2% in men and 88.2% in women, with recalibration failing to improve performance, as underestimation rates remained above 80% for both sexes.^23^ A study involving 3,347 participants aged 40 to 79 years in Xinjiang, a multiethnic rural area, revealed that the PCE significantly underestimated 5-year ASCVD risk in both women and men, and recalibration did not change discrimination.^11^ Notably, both our study and the CHERRY (CHinese Electronic health Records Research in Yinzhou) study, which included 226,406 participants aged 40 to 79 years in Yinzhou, a developed area of southeastern China, reported substantial improvement in overall calibration after recalibrating to local population, although discrepancies persisted in the highest-risk groups.^18^

The China-PAR model has been adopted as the ASCVD risk calculator for primary prevention by the Chinese guidelines in 2019.^37^ However, multiple external validation studies revealed consistent misestimated of ASCVD risk by China-PAR model. In the CIMIC (Community Intervention of Metabolic Syndrome in China and Chinese Family Health Study) cohort, the China-PAR equations overestimated the 5-year ASCVD risk by 11.9% in men and 27.5% in women compared with observed outcomes.^17^ In the CHERRY study, China-PAR underestimated 5-year ASCVD risk by 20% for men and 40% for women.^18^ Similar to our previous findings in a Northern Chinese population that China-PAR overestimated 5-year ASCVD risk by 29.4% risk in women.^23^ To address the issue of overestimating 10-year ASCVD risk in our local population, the China-PAR needs recalibration to enhance predictive accuracy, thereby better guiding clinical decisions and ensuring appropriate interventions. In our study, the recalibrated China-PAR demonstrated improved alignment between predicted and observed risks, particularly in lower-risk groups. Although predicted 10-year ASCVD risk in the high-risk group remained either overestimated or underestimated, the overall calibration was enhanced, thereby reducing prediction bias.

The PREVENT equations, a novel cardiovascular risk estimation tool for the general population, have been validated across diverse multiethnic populations.^38^, ^39^, ^40^, ^41^ For example, in the VHA (Veterans Health Administration) cohort, the PREVENT equations demonstrated modest overall predictive performance (overall C-index: 0.634) among the veteran population, with variability across racial and ethnic groups (C-indexes ranged from 0.624 to 0.681).^38^ In an analysis of electronic health records from 270,320 patients in the United States, the PREVENT equations also demonstrated heterogeneous calibration and discrimination performance across sex, race and ethnicity, and sociodemographics.^39^ To the best of our knowledge, the PREVENT equations have not yet been validated in the Chinese population. Our findings further indicated that the model adequately predicted risk in women but underestimated the 10-year ASCVD risk in men, which was line with previous studies.^38^^,^^39^ Consistent with prior studies that recalibrated the PREVENT model using complex statistical methodologies, our application of a straightforward recalibration approach resulted in a significant improvement in the model’s calibration performance.^38^^,^^42^ Although the PREVENT equations demonstrated modest-to-good discriminative performance in the present population, the addition of specific biomarkers of insulin resistance and inflammation to the model did not improve its discriminative ability. Further large-scale cohort studies are needed to explore novel biomarkers for enhancing discriminative performance.

In practical clinical settings, insufficient individual participant data for creating new models is the reason that many clinicians lack the means to access customized models for every distinct population. The population-based recalibration of ASCVD risk prediction model offers a practical and feasible approach to enhancing cardiovascular risk assessment. The recommendation to tailor existing models, rather than developing new ones for diverse populations, is supported by the substantial improvement observed in the recalibrated PCE and PREVENT models.^12^^,^^18^^,^^38^^,^^42^^,^^43^ One of the advantages of recalibrating the PCE, China-PAR, and PREVENT models is its ability to provide a compromise in general risk assessment for primary prevention in clinical practice. Given the widespread acceptance and use of the PCE, China-PAR, and PREVENT models, recalibration ensures continuity and familiarity for clinicians. Moreover, by utilizing the relatively smaller local data sets, the recalibrated ASCVD risk prediction model can be adapted to diverse populations, which is a more efficient approach, particularly important in resource-limited settings where challenging to access comprehensive, high-quality data. Additionally, recalibrating the ASCVD risk prediction model remains the robustness of the original model, which is built on extensive and diverse epidemiological data, ensuring that the recalibrated model retains its foundational strengths while improving its applicability to new populations.

Despite the improvements achieved through recalibrating the ASCVD risk prediction model, attention on individuals in the highest-risk groups is crucial, because they are more likely to experience adverse cardiovascular events and benefit most from precise risk stratification and targeted preventive measures. The inclusion of risk-enhancing factors and coronary artery calcium scores, as well as the integration of polygenic risk scores for coronary artery disease, has been recommended to improve risk stratification and reclassify individuals with borderline or intermediate risk to higher-risk categories.^44^^,^^45^ Furthermore, using advanced statistical methods, such as constrained optimization for recalibration, can enhance the net benefit of risk models by ensuring better calibration around critical risk thresholds.^46^ Recalibration should not only align predicted and observed risks but also improve risk prediction accuracy for those at the highest risk by considering additional data sources, such as genetic and biochemical markers. For instance, biomarkers indicating inflammation or endothelial dysfunction can offer additional risk information,^47^^,^^48^ which could contribute to identifying individuals who might otherwise be classified as lower risk.

### Study strengths and limitations

One of its primary strengths is the large sample size from the Kailuan study, which includes 79,497 participants. Additionally, the significant improvement in the predictive performance of recalibrated PCE, China-PAR, and PREVENT models indicates that it could be better suited for the Chinese population compared with the original model. This study supplements the limited evidence currently available on the recalibration of PCE, China-PAR, and PREVENT models in the Chinese population and confirms the usability of PCE and PREVENT models compared with China-PAR model in this context. However, the study also has several limitations. First, a major limitation stemmed from the sex imbalance, with a higher proportion of male participants, which may potentially compromise the generalizability of the findings to the general population, particularly women. Second, as an observational study, missing data and extreme values were inevitable. We employed the multiple imputation by chained equations algorithm and Winsorization method to address these issues in the analyses. Third, in the current analysis, ASCVD events only included myocardial infarction and ischemic stroke, which may have been underascertained in the study population. Fourth, this study focused on the PREVENT base equations, whereas the PREVENT full equations were not evaluated herein caused by the lack of key predictors, including the urine albumin-to-creatinine ratio, hemoglobin A1c, and social deprivation index.

## Conclusions

This cohort study provided a comprehensive assessment of the PCE, China-PAR, and PREVENT models in a large contemporary Chinese population. When applied to diverse populations, these original ASCVD primary prevention risk scores may yield inaccurate estimates of ASCVD risk. Recalibration to the local population may improve performance of both the PCE, China-PAR, and PREVENT models for general ASCVD risk assessment.

## Funding Support and Author Disclosures

This work was supported by the Capital’s Funds for Health Improvement and Research (CFH2024-2G-2036); the National Natural Science Foundation of China (72474142 and 82103942); the Beijing Nova Program (20250484858); the R&D Program of Beijing Municipal Education Commission (KM202210025015); the Clinical Research Incubation Project, Beijing Chao-Yang Hospital, Capital Medical University (CYFH202310), and the Talent development plan for the future in Medical-Engineering Integration by BRA-CDCHE and ZTA (MBRC0012025021). The authors have reported that they have no relationships relevant to the contents of this paper to disclose.
