# Supplementary material for: Validation and Recalibration of PCE, China-PAR, and PREVENT Models for Estimating ASCVD Risk in China
Source: JACC Asia. 2026 Feb 20;6(5):602–16. doi: 10.1016/j.jacasi.2025.12.015 (PMC13153916; doi:10.1016/j.jacasi.2025.12.015)
Supplement: Supplemental Material [file mmc1.docx]

**Supplemental Methods**

**Multiple imputation**

The missingness percentages of covariates ranged from 1.24% to 3.59%. We first plotted the missing data pattern and then applied Little's Missing Completely at Random (MCAR) test to the dataset to determine whether the data were missing completely at random. We hypothesized that the missingness in covariates was missing at random (MAR) if not completely at random. We performed multiple imputation by chained equations (MICE) to address the missing data. We opted for Predictive Mean Matching (PMM) to impute continuous variables and a logistic model for binary variables. In the imputation model, we included the baseline covariates, Nelson-Aalen estimate of the cumulative hazard function of ASCVD, and ASCVD status. We produced 5 imputed data sets, and the estimates from each imputed data set were combined into one overall estimate with the use of Rubin’s rule. We compared the distribution of the imputed data with that of the complete data.

To test the robustness of the imputation, we conducted a complete-case analysis and compared results with the main analyses. For instance, the Harrell’s C-index for females in the main analysis was calculated at 0.735 (95% CI: 0.712 to 0.757). A subsequent complete case-analysis produced a highly similar C-index of 0.734 (95% CI: 0.711 to 0.758). This demonstrates that the estimates were consistent with those derived from the main analysis.

References:

1. van Buuren S, Groothuis-Oudshoorn K. mice: Multivariate Imputation by Chained Equations in R. J. Stat. Soft. 2011;45(3):1-6
2. White IR, Royston P, Wood AM. Multiple imputation using chained equations: Issues and guidance for practice. Stat Med. 2011;30(4):377-399.

**(A) Missing data pattern**


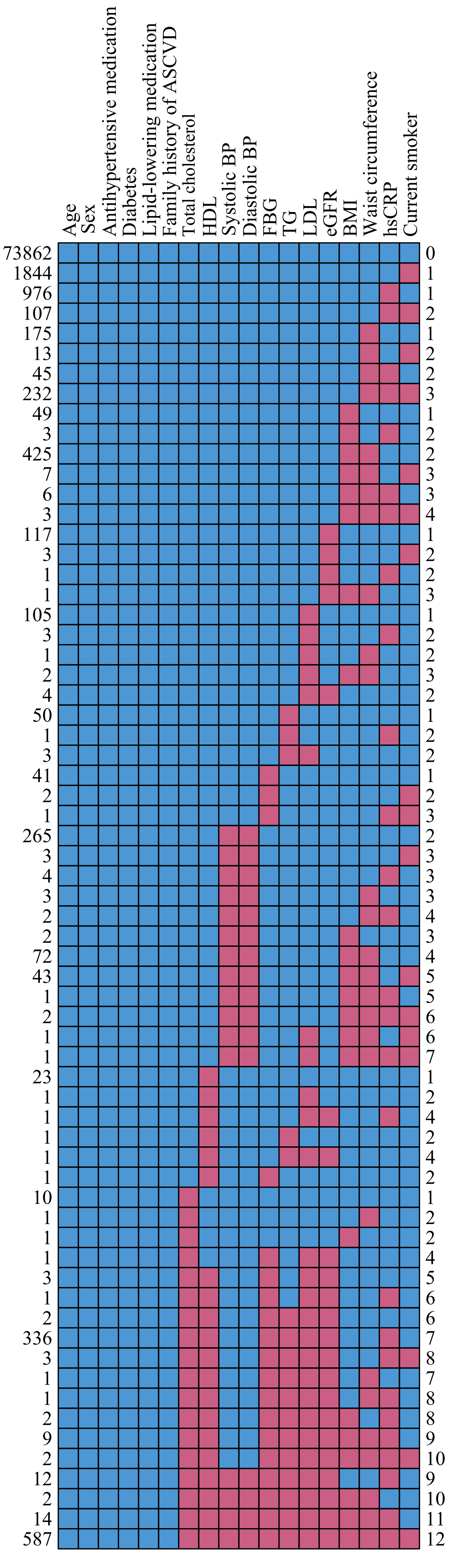


**(B)** Distribution of the imputed data


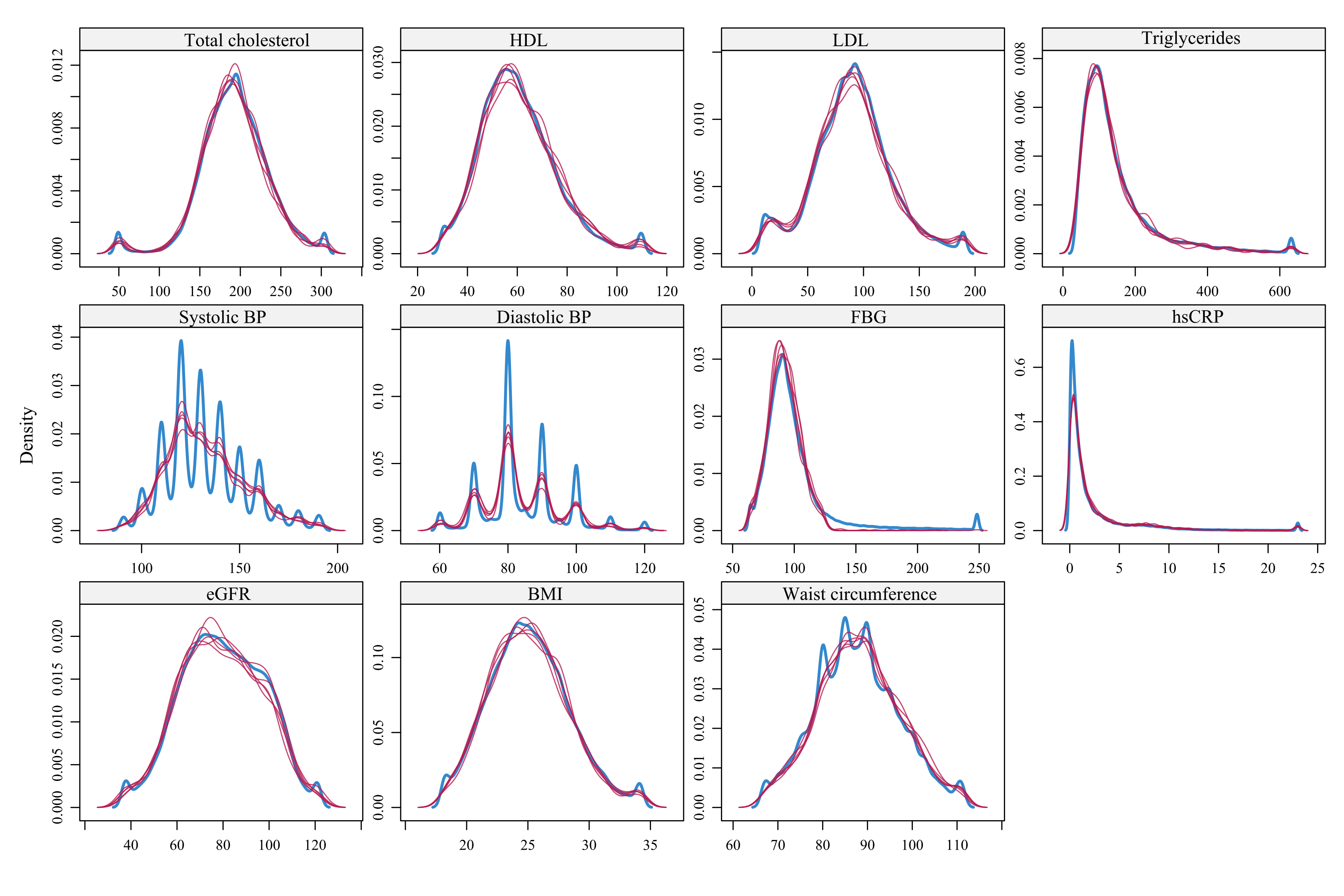


| **Supplemental Table 1.** Number and Percentage of Missing Data | | |
| --- | --- | --- |
|  | n | % |
| Current smoker | 2854 | 3.59 |
| hsCRP | 2356 | 2.96 |
| Waist circumference | 1652 | 2.08 |
| BMI | 1235 | 1.55 |
| eGFR | 1104 | 1.39 |
| LDL | 1099 | 1.38 |
| Triglycerides | 1027 | 1.29 |
| FBG | 1021 | 1.28 |
| Systolic BP | 1014 | 1.28 |
| Diastolic BP | 1014 | 1.28 |
| HDL | 1003 | 1.26 |
| Total cholesterol | 988 | 1.24 |
| Abbreviations as in Table 1. | | |

| **Supplemental Table 2. Equations for 10-Year ASCVD Risk Using Original PCE, China-PAR, and PRVENT (Base) Model** | | | |
| --- | --- | --- | --- |
| **Model** | **S0(t) at 10 years** | **Mean score** | **Equations for Individual Score** |
| **PCE (Caucasian)** |  |  | Equations for PCE (Caucasian) including S_0_(t) at 10-year were obtained from the Supplementary Table S1 in JAMA 2014; 311:1406–1415.  Final risk estimation was calculated as: Predicted ASCVD risk = 1 - S_0_(t)^exp(Individual score – Mean score). |
| Men | 0.9144 | 61.18 | =12.344×ln(age)+11.853×ln(TC)-2.664×ln(age)×ln(TC)-7.990×ln(HDL-C)+1.769×ln(age)×ln(HDL-C) (+1.797×ln(SBP) if hypertension treated) (+1.764×ln(SBP) if hypertension untreated) (+7.837-1.795×ln(age) if current smoker) (+0.658 if diabetes) |
| Women | 0.9665 | -29.18 | =-29.799×ln(age)+4.884×ln(age)×ln(age)+13.540×ln(TC)-3.114×ln(age)×ln(TC)-13.578×ln(HDL-C) +3.149×ln(age)×ln(HDL-C) (+2.019×ln(SBP) if hypertension treated) (+1.957×ln(SBP) if hypertension untreated) (+7.574-1.665×ln(age) if current smoker) (+0.661 if diabetes) |
| **China-PAR** |  |  | Equations for China-PAR including S_0_(t) at 10-year were obtained from the Supplementary Table S1 in Circulation 2016;134(19):1430–1440 and Environ Sci Technol 2023;57(27):9934–9942.  Final risk estimation was calculated as: Predicted ASCVD risk = 1 - S_0_(t)^exp(Individual score – Mean score).  *Note: All participants in the Kailuan cohort are presumed to reside in urban areas of Northern China. |
| Men | 0.9707 | 140.68 | =31.97×ln(age)+0.62×ln(TC)-0.69×ln(HDL-C)-0.71×ln(waist) (+[27.39-6.02×ln(age)] ×ln(SBP) if hypertension treated) (+[26.15-5.73×ln(age)]×ln(SBP) if hypertension untreated) (+3.96-0.94×ln(age) if current smoker) (+6.22-1.53×ln(age) if having family history of ASCVD) (+0.36 if diabetes) (+0.48 if in Northern China) (-0.16 if living in urban) |
| Women | 0.9851 | 117.26 | =24.87×ln(age)+0.06×ln(TC)-0.22×ln(HDL-C)+1.48×ln(waist) (+[20.71-4.53×ln(age)] ×ln(SBP) if hypertension treated) (+[19.98 -4.36×ln(age)]×ln(SBP) if hypertension untreated) (+0.49 if current smoker) (+0.57 if diabetes) (+0.54 if in Northern China) |
| **PRVENT (base)** |  |  | Equations for PRVENT-ASCVD (base) were obtained from the Supplementary Appendix 4 in Circulation 2024;149(6):430–449.  Final risk estimation was calculated as: Predicted ASCVD risk = exp(log-Odds) / (1 + exp(log-Odds)).  *Note: Participants in the Kailuan cohort who reported using lipid-lowering medication are presumed to have been taking statins. |
| Men |  |  | log-Odds = – 3.500655 + 0.7099847 × (age – 55) /10 + 0.1658663 × ((TC – HDL) × 0.02586 – 3.5) – 0.1144285 × (HDL × 0.02586 – 1.3) /0.3 – 0.2837212 × (min(SBP, 110) – 110) /20 + 0.3239977 × (max(SBP, 110) – 130) /20 + 0.7189597 × (if diabetes) + 0.3956973 × (if current smoker) + 0.3690075 × (min(eGFR, 60) – 60) / -15 + 0.0203619 × (max(eGFR, 60) – 90) / -15 + 0.2036522 × (if using anti-hypertensive medication) – 0.0865581 × (if using statin) – 0.0322916 × (if using anti-hypertensive medication) × (max(SBP, 110) – 130) /20 + 0.114563 × (if using statin) × ((TC – HDL) × 0.02586 – 3.5) – 0.0300005 × (age – 55) /10 × ((TC – HDL) × 0.02586 – 3.5) + 0.0232747 × (age – 55) /10 × (HDL × 0.02586 – 1.3) /0.3 – 0.0927024 × (age – 55) /10 × (max(SBP, 110) – 130) /20 – 0.2018525 × (age – 55) /10 × (if diabetes) – 0.0970527 × (age – 55) /10 × (if current smoker) – 0.1217081  × (age – 55) /10 × (min(eGFR, 60) – 60) / -15 |
| Women |  |  | log-Odds = -3.819975 + 0.719883 × (age – 55) /10 + 0.1176967 × ((TC – HDL) × 0.02586 – 3.5) – 0.151185 × (HDL × 0.02586 – 1.3) /0.3 – 0.0835358 × (min(SBP, 110) – 110) /20 + 0.3592852 × (max(SBP, 110) – 130) /20 + 0.8348585 × (if diabetes) + 0.4831078 × (if current smoker) + 0.4864619 × (min(eGFR, 60) – 60) / -15 + 0.0397779 × (max(eGFR, 60) – 90) / -15 + 0.2265309 × (if using anti-hypertensive medication) – 0.0592374 × (if using statin) – 0.0395762 × (if using anti-hypertensive medication) × (max(SBP, 110) – 130) /20 + 0.0844423 × (if using statin) × ((TC – HDL) × 0.02586 – 3.5) – 0.0567839 × (age – 55) /10 × ((TC – HDL) × 0.02586 – 3.5) + 0.0325692 × (age – 55) /10 × (HDL × 0.02586 – 1.3) /0.3 – 0.1035985 × (age – 55) /10 × (max(SBP, 110) – 130) /20 – 0.2417542 × (age – 55) /10 × (if diabetes) – 0.0791142 × (age – 55) /10 × (if current smoker) – 0.1671492 × (age – 55) /10 × (min(eGFR, 60) – 60) / -15 |
| ASCVD, atherosclerotic cardiovascular disease; China-PAR, Prediction of atherosclerotic CVD risk in China; eGFR, estimated glomerular ﬁltration rate; PCE, Pooled Cohort Equation;  PREVENT, Predicting Risk of Cardiovascular Disease EVENTs; SBP, systolic blood pressure; TC, total cholesterol; HDL-C, high-density lipoprotein cholesterol. | | | |

| **Supplemental Table 3. Equations for 10-Year ASCVD Risk using Recalibrated PCE, China-PAR, and PRVENT (Base) Model (Method 1)** | | | |
| --- | --- | --- | --- |
| **Model** | **S0(t) at 10 years*** | **Mean score*** | **Equations for Individual Score** |
| **PCE (Caucasian)** |  |  | Predicted ASCVD risk = 1 – S_0_(t)^exp(Individual score – Mean score). |
| Men | 0.9557 | 60.93 | Individual score = 12.344×ln(age)+11.853×ln(TC)-2.664×ln(age)×ln(TC)-7.990×ln(HDL-C)+1.769×ln(age)×ln(HDL-C) (+1.797×ln(SBP) if hypertension treated) (+1.764×ln(SBP) if hypertension untreated) (+7.837-1.795×ln(age) if current smoker) (+0.658 if diabetes) |
| Women | 0.9863 | -29.94 | Individual score = -29.799×ln(age)+4.884×ln(age)×ln(age)+13.540×ln(TC)-3.114×ln(age)×ln(TC)-13.578×ln(HDL-C) +3.149×ln(age)×ln(HDL-C) (+2.019×ln(SBP) if hypertension treated) (+1.957×ln(SBP) if hypertension untreated) (+7.574-1.665×ln(age) if current smoker) (+0.661 if diabetes) |
| **China-PAR** |  |  | Predicted ASCVD risk = 1 - S_0_(t)^exp(Individual score – Mean score). |
| Men | 0.9502 | 141.39 | Individual score = 31.97×ln(age)+0.62×ln(TC)-0.69×ln(HDL-C)-0.71×ln(waist) (+[27.39-6.02×ln(age)] ×ln(SBP) if hypertension treated) (+[26.15-5.73×ln(age)]×ln(SBP) if hypertension untreated) (+3.96-0.94×ln(age) if current smoker) (+6.22-1.53×ln(age) if having family history of ASCVD) (+0.36 if diabetes) (+0.48 if in Northern China) (-0.16 if living in urban) |
| Women | 0.9809 | 118.17 | Individual score = 24.87×ln(age)+0.06×ln(TC)-0.22×ln(HDL-C)+1.48×ln(waist) (+[20.71-4.53×ln(age)] ×ln(SBP) if hypertension treated) (+[19.98 -4.36×ln(age)]×ln(SBP) if hypertension untreated) (+0.49 if current smoker) (+0.57 if diabetes) (+0.54 if in Northern China) |
| **PRVENT (base)** | β_0_ | α | Predicted ASCVD risk = exp(log-Odds) / (1 + exp(log-Odds)). |
| Men | 0.2296 | NA | log-Odds = **0.22917** – 3.500655 + 0.7099847 × (age – 55) /10 + 0.1658663 × ((TC – HDL) × 0.02586 – 3.5) – 0.1144285 × (HDL × 0.02586 – 1.3) /0.3 – 0.2837212 × (min(SBP, 110) – 110) /20 + 0.3239977 × (max(SBP, 110) – 130) /20 + 0.7189597 × (if diabetes) + 0.3956973 × (if current smoker) + 0.3690075 × (min(eGFR, 60) – 60) / -15 + 0.0203619 × (max(eGFR, 60) – 90) / -15 + 0.2036522 × (if using anti-hypertensive medication) – 0.0865581 × (if using statin) – 0.0322916 × (if using anti-hypertensive medication) × (max(SBP, 110) – 130) /20 + 0.114563 × (if using statin) × ((TC – HDL) × 0.02586 – 3.5) – 0.0300005 × (age – 55) /10 × ((TC – HDL) × 0.02586 – 3.5) + 0.0232747 × (age – 55) /10 × (HDL × 0.02586 – 1.3) /0.3 – 0.0927024 × (age – 55) /10 × (max(SBP, 110) – 130) /20 – 0.2018525 × (age – 55) /10 × (if diabetes) – 0.0970527 × (age – 55) /10 × (if current smoker) – 0.1217081 × (age – 55) /10 × (min(eGFR, 60) – 60) / -15 |
| Women | 0.0232 | NA | log-Odds = **0.02222** –3.819975 + 0.719883 × (age – 55) /10 + 0.1176967 × ((TC – HDL) × 0.02586 – 3.5) – 0.151185 × (HDL × 0.02586 – 1.3) /0.3 – 0.0835358 × (min(SBP, 110) – 110) /20 + 0.3592852 × (max(SBP, 110) – 130) /20 + 0.8348585 × (if diabetes) + 0.4831078 × (if current smoker) + 0.4864619 × (min(eGFR, 60) – 60) / -15 + 0.0397779 × (max(eGFR, 60) – 90) / -15 + 0.2265309 × (if using anti-hypertensive medication) – 0.0592374 × (if using statin) – 0.0395762 × (if using anti-hypertensive medication) × (max(SBP, 110) – 130) /20 + 0.0844423 × (if using statin) × ((TC – HDL) × 0.02586 – 3.5) – 0.0567839 × (age – 55) /10 × ((TC – HDL) × 0.02586 – 3.5) + 0.0325692 × (age – 55) /10 × (HDL × 0.02586 – 1.3) /0.3 – 0.1035985 × (age – 55) /10 × (max(SBP, 110) – 130) /20 – 0.2417542 × (age – 55) /10 × (if diabetes) – 0.0791142 × (age – 55) /10 × (if current smoker) – 0.1671492 × (age – 55) /10 × (min(eGFR, 60) – 60) / -15 |
| ASCVD, atherosclerotic cardiovascular disease; China-PAR, Prediction of atherosclerotic CVD risk in China; eGFR, estimated glomerular ﬁltration rate; PCE, Pooled Cohort Equation;  PREVENT, Predicting Risk of Cardiovascular Disease EVENTs; SBP, systolic blood pressure; TC, total cholesterol; HDL-C, high-density lipoprotein cholesterol.  *We produced 5 imputed data sets, and the estimates from each imputed data set were combined into one overall estimate with the use of Rubin’s rule. | | | |

| **Supplemental Table 4. Equations for 10-Year ASCVD Risk using Recalibrated PCE, China-PAR, and PRVENT (Base) Model (Method 2)** | | | |
| --- | --- | --- | --- |
| **Model** | **S0(t) at 10 years*** | **Mean score*** | **Equations for Individual Score** |
| **PCE (Caucasian)** |  |  | Predicted ASCVD risk = 1 – S_0_(t)^exp(Individual score – Mean score). |
| Men | 0.9452 | 60.93×0.6516 | Individual score = **0.6516**×[12.344×ln(age)+11.853×ln(TC)-2.664×ln(age)×ln(TC)-7.990×ln(HDL-C)+1.769×ln(age)×ln(HDL-C) (+1.797×ln(SBP) if hypertension treated) (+1.764×ln(SBP) if hypertension untreated) (+7.837-1.795×ln(age) if current smoker) (+0.658 if diabetes)] |
| Women | 0.9792 | -29.94×0.6721 | Individual score = **0.6721**×[-29.799×ln(age)+4.884×ln(age)×ln(age)+13.540×ln(TC)-3.114×ln(age)×ln(TC)-13.578×ln(HDL-C) +3.149×ln(age)×ln(HDL-C) (+2.019×ln(SBP) if hypertension treated) (+1.957×ln(SBP) if hypertension untreated) (+7.574-1.665×ln(age) if current smoker) (+0.661 if diabetes)] |
| **China-PAR** |  |  | Predicted ASCVD risk = 1 - S_0_(t)^exp(Individual score – Mean score). |
| Men | 0.9465 | 141.39×0.8197 | Individual score = **0.8197**×[31.97×ln(age)+0.62×ln(TC)-0.69×ln(HDL-C)-0.71×ln(waist) (+[27.39-6.02×ln(age)] ×ln(SBP) if hypertension treated) (+[26.15-5.73×ln(age)]×ln(SBP) if hypertension untreated) (+3.96-0.94×ln(age) if current smoker) (+6.22-1.53×ln(age) if having family history of ASCVD) (+0.36 if diabetes) (+0.48 if in Northern China) (-0.16 if living in urban)] |
| Women | 0.9804 | 118.17×0.9582 | Individual score = **0.9582**×[24.87×ln(age)+0.06×ln(TC)-0.22×ln(HDL-C)+1.48×ln(waist) (+[20.71-4.53×ln(age)] ×ln(SBP) if hypertension treated) (+[19.98 -4.36×ln(age)]×ln(SBP) if hypertension untreated) (+0.49 if current smoker) (+0.57 if diabetes) (+0.54 if in Northern China)] |
| **PRVENT (base)** | β_0_ | α | Predicted ASCVD risk = exp(log-Odds) / (1 + exp(log-Odds)). |
| Men | -0.3206 | 0.7987 | log-Odds = **-0.3206** + **0.7987** × [– 3.500655 + 0.7099847 × (age – 55) /10 + 0.1658663 × ((TC – HDL) × 0.02586 – 3.5) – 0.1144285 × (HDL × 0.02586 – 1.3) /0.3 – 0.2837212 × (min(SBP, 110) – 110) /20 + 0.3239977 × (max(SBP, 110) – 130) /20 + 0.7189597 × (if diabetes) + 0.3956973 × (if current smoker) + 0.3690075 × (min(eGFR, 60) – 60) / -15 + 0.0203619 × (max(eGFR, 60) – 90) / -15 + 0.2036522 × (if using anti-hypertensive medication) – 0.0865581 × (if using statin) – 0.0322916 × (if using anti-hypertensive medication) × (max(SBP, 110) – 130) /20 + 0.114563 × (if using statin) × ((TC – HDL) × 0.02586 – 3.5) – 0.0300005 × (age – 55) /10 × ((TC – HDL) × 0.02586 – 3.5) + 0.0232747 × (age – 55) /10 × (HDL × 0.02586 – 1.3) /0.3 – 0.0927024 × (age – 55) /10 × (max(SBP, 110) – 130) /20 – 0.2018525 × (age – 55) /10 × (if diabetes) – 0.0970527 × (age – 55) /10 × (if current smoker) – 0.1217081 × (age – 55) /10 × (min(eGFR, 60) – 60) / -15] |
| Women | -0.2979 | 0.8991 | log-Odds = **-0.2979** + **0.8991** × [–3.819975 + 0.719883 × (age – 55) /10 + 0.1176967 × ((TC – HDL) × 0.02586 – 3.5) – 0.151185 × (HDL × 0.02586 – 1.3) /0.3 – 0.0835358 × (min(SBP, 110) – 110) /20 + 0.3592852 × (max(SBP, 110) – 130) /20 + 0.8348585 × (if diabetes) + 0.4831078 × (if current smoker) + 0.4864619 × (min(eGFR, 60) – 60) / -15 + 0.0397779 × (max(eGFR, 60) – 90) / -15 + 0.2265309 × (if using anti-hypertensive medication) – 0.0592374 × (if using statin) – 0.0395762 × (if using anti-hypertensive medication) × (max(SBP, 110) – 130) /20 + 0.0844423 × (if using statin) × ((TC – HDL) × 0.02586 – 3.5) – 0.0567839 × (age – 55) /10 × ((TC – HDL) × 0.02586 – 3.5) + 0.0325692 × (age – 55) /10 × (HDL × 0.02586 – 1.3) /0.3 – 0.1035985 × (age – 55) /10 × (max(SBP, 110) – 130) /20 – 0.2417542 × (age – 55) /10 × (if diabetes) – 0.0791142 × (age – 55) /10 × (if current smoker) – 0.1671492 × (age – 55) /10 × (min(eGFR, 60) – 60) / -15] |
| ASCVD, atherosclerotic cardiovascular disease; China-PAR, Prediction of atherosclerotic CVD risk in China; eGFR, estimated glomerular ﬁltration rate; PCE, Pooled Cohort Equation;  PREVENT, Predicting Risk of Cardiovascular Disease EVENTs; SBP, systolic blood pressure; TC, total cholesterol; HDL-C, high-density lipoprotein cholesterol.  *We produced 5 imputed data sets, and the estimates from each imputed data set were combined into one overall estimate with the use of Rubin’s rule. | | | |

| **Supplemental Table 5. Baseline Characteristics by Sex** | | |
| --- | --- | --- |
|  | **Women**  **(n = 15652)** | **Men**  **(n = 63845)** |
| Age, mean (SD), y | 52.73 (8.24) | 55.28 (9.05) |
| Current smoker, n (%) | 314 (2.1) | 25719 (41.8) |
| Diabetes mellitus, n (%) | 1479 (9.4) | 6649 (10.4) |
| Antihypertensive medication, n (%) | 2036 (13.0) | 6899 (10.8) |
| Lipid-lowering medication, n (%) | 190 (1.2) | 464 (0.7) |
| Family history of ASCVD, n (%) | 1199 (7.7) | 3491 (5.5) |
| Blood pressure, mean (SD), mm Hg |  |  |
| Systolic | 128.02 (20.59) | 133.86 (20.26) |
| Diastolic | 80.98 (10.85) | 85.20 (11.53) |
| BMI, mean (SD), kg/m^2^ | 25.05 (3.51) | 25.13 (3.23) |
| Waist circumference, mean (SD), cm | 84.70 (9.67) | 88.39 (9.04) |
| Cholesterol, mean (SD), mmol/L |  |  |
| Total | 5.07 (1.05) | 4.98 (1.08) |
| HDL | 1.61 (0.39) | 1.55 (0.39) |
| LDL | 2.22 (0.90) | 2.36 (0.89) |
| Triglycerides, mean (SD), mmol/L | 1.27 [0.90, 1.87] | 1.30 [0.92, 1.99] |
| FBG, mean (SD), mmol/L | 5.38 (1.60) | 5.56 (1.60) |
| hsCRP, median [IQR], mg/L | 0.96 [0.31, 2.73] | 0.80 [0.30, 2.20] |
| eGFR, median [IQR], mL/min/1.73 m^2^ | 75.89 [64.14, 89.58] | 80.52 [67.75, 94.45] |
| TyG index, mean (SD) | 8.62 (0.65) | 8.70 (0.68) |
| 10-y incident ASCVD | 437 (2.8) | 3988 (6.2) |
| PCE 10-y ASCVD risk, median [IQR], % | 1.37 [0.66, 3.17] | 6.77 [3.50, 13.03] |
| China-PAR 10-y ASCVD risk, median [IQR], % | 3.59 [1.79, 7.37] | 5.90 [3.22, 10.83] |
| PREVENT 10-y ASCVD risk, median [IQR], % | 1.63 [0.86, 3.44] | 3.64 [2.12, 6.65] |

| **Supplemental Table 6. Baseline Characteristics in the Derivation Set and Validation Set** | | |
| --- | --- | --- |
|  | **Derivation**  **(n = 47940)** | **Validation**  **(n = 31557)** |
| Age, mean (SD), y | 54.78 (8.94) | 54.78 (8.96) |
| Men, n (%) | 38491 (80.3) | 25354 (80.3) |
| Current smoker, n (%) | 15696 (34.0) | 10337 (34.0) |
| Diabetes mellitus, n (%) | 4939 (10.3) | 3189 (10.1) |
| Antihypertensive medication, n (%) | 5390 (11.2) | 3545 (11.2) |
| Lipid-lowering medication, n (%) | 415 (0.9) | 239 (0.8) |
| Family history of ASCVD, n (%) | 2844 (5.9) | 1846 (5.8) |
| Blood pressure, mean (SD), mm Hg |  |  |
| Systolic | 132.71 (20.49) | 132.67 (20.38) |
| Diastolic | 84.34 (11.52) | 84.41 (11.53) |
| BMI, mean (SD), kg/m^2^ | 25.12 (3.29) | 25.10 (3.28) |
| Waist circumference, mean (SD), cm | 87.67 (9.31) | 87.66 (9.25) |
| Cholesterol, mean (SD), mmol/L |  |  |
| Total | 5.00 (1.08) | 4.99 (1.08) |
| HDL | 1.56 (0.39) | 1.56 (0.39) |
| LDL | 2.33 (0.89) | 2.34 (0.89) |
| Triglycerides, mean (SD), mmol/L | 1.30 [0.92, 1.96] | 1.29 [0.92, 1.96] |
| FBG, mean (SD), mmol/L | 5.53 (1.61) | 5.52 (1.59) |
| hsCRP, median [IQR], mg/L | 0.83 [0.30, 2.31] | 0.84 [0.30, 2.30] |
| eGFR, median [IQR], mL/min/1.73 m^2^ | 79.61 [67.02, 93.70] | 79.44 [66.94, 93.55] |
| TyG index, mean (SD) | 8.69 (0.68) | 8.68 (0.67) |
| 10-y incident ASCVD | 2654 (5.5) | 1771 (5.6) |
| PCE 10-y ASCVD risk, median [IQR], % | 5.50 [2.35, 11.43] | 5.52 [2.30, 11.44] |
| China-PAR 10-y ASCVD risk, median [IQR], % | 3.22 [1.78, 6.09] | 3.24 [1.76, 6.07] |
| PREVENT 10-y ASCVD risk, median [IQR], % | 5.41 [2.87, 10.17] | 5.46 [2.83, 10.13] |

| **Supplemental Table 7.** Association of Biomarkers **(**Tyg index and hsCRP) and Risk of ASCVD In Whole Cohort, After Adjusting for Original PCE, China-PAR, And PREVENT Risk Score | | | |
| --- | --- | --- | --- |
|  | HR (95% CI) | | |
|  | + Original PCE | + Original China-PAR | + Original PREVENT |
| **Women (n = 15652)** |  |  |  |
| TyG index, per SD | 1.26 (1.15-1.39) | 1.19 (1.08-1.31) | 1.16 (1.05-1.28) |
| hsCRP (ln), per SD | 1.14 (1.03-1.27) | 1.11 (1.00-1.23) | 1.15 (1.04-1.28) |
| **Men (n = 63845)** |  |  |  |
| TyG index, per SD | 1.15 (1.11-1.18) | 1.14 (1.11-1.18) | 1.10 (1.07-1.13) |
| hsCRP (ln), per SD | 1.19 (1.15-1.23) | 1.20 (1.16-1.24) | 1.19 (1.15-1.23) |

**(A) PCE score + TyG index + hsCRP**


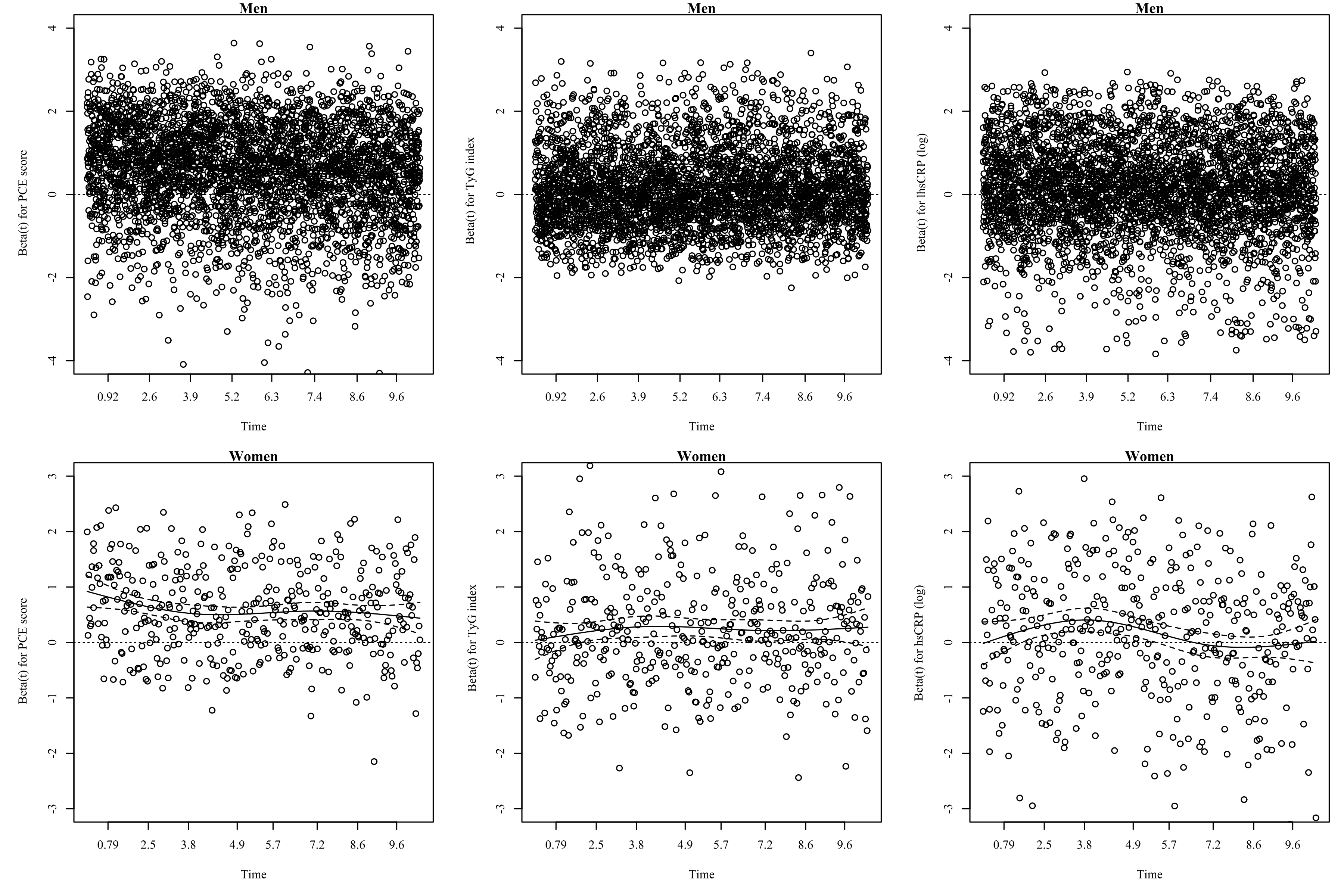


**(B) China-PAR score + TyG index + hsCRP**


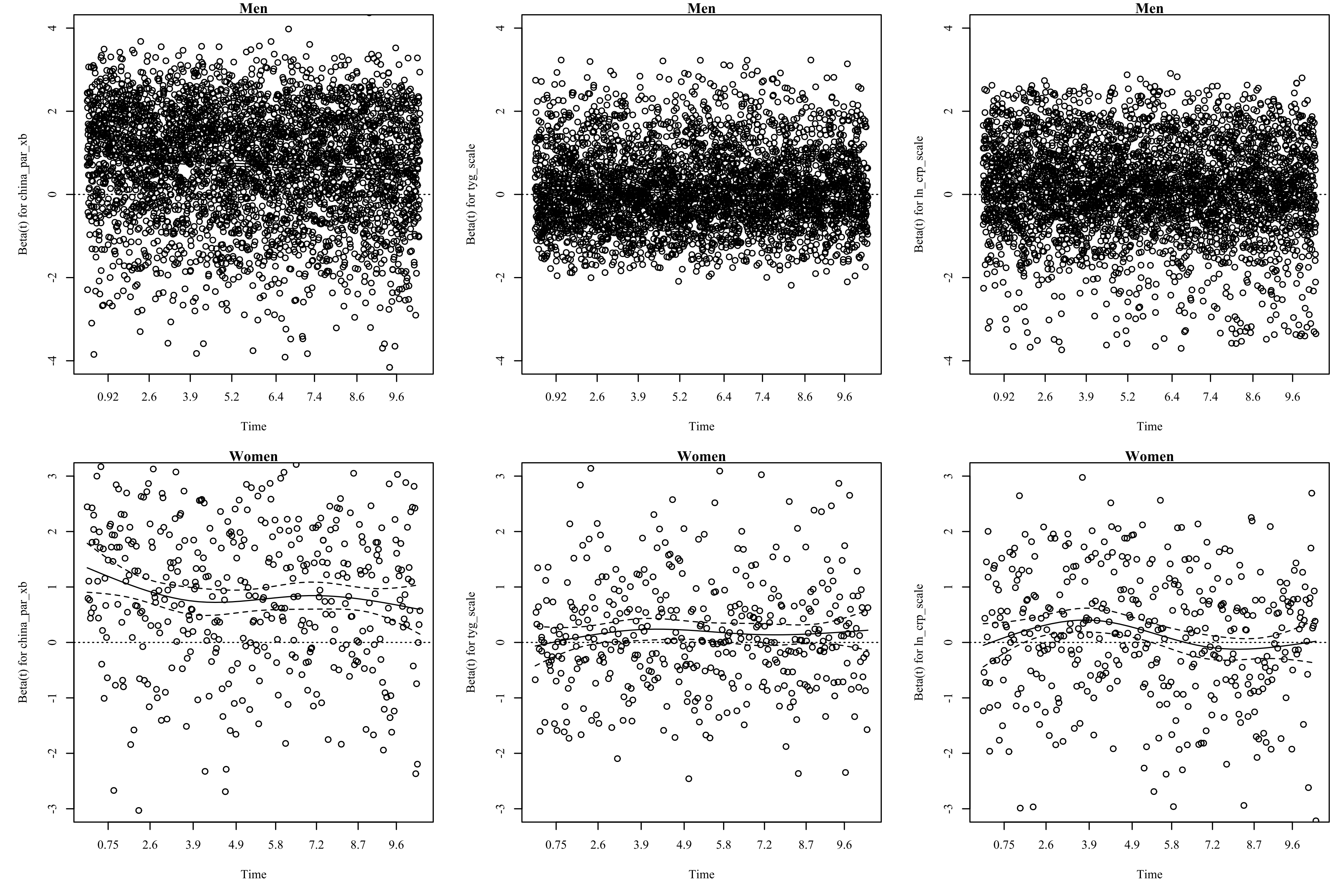


**(C)** **PREVENT score + TyG index + hsCRP**


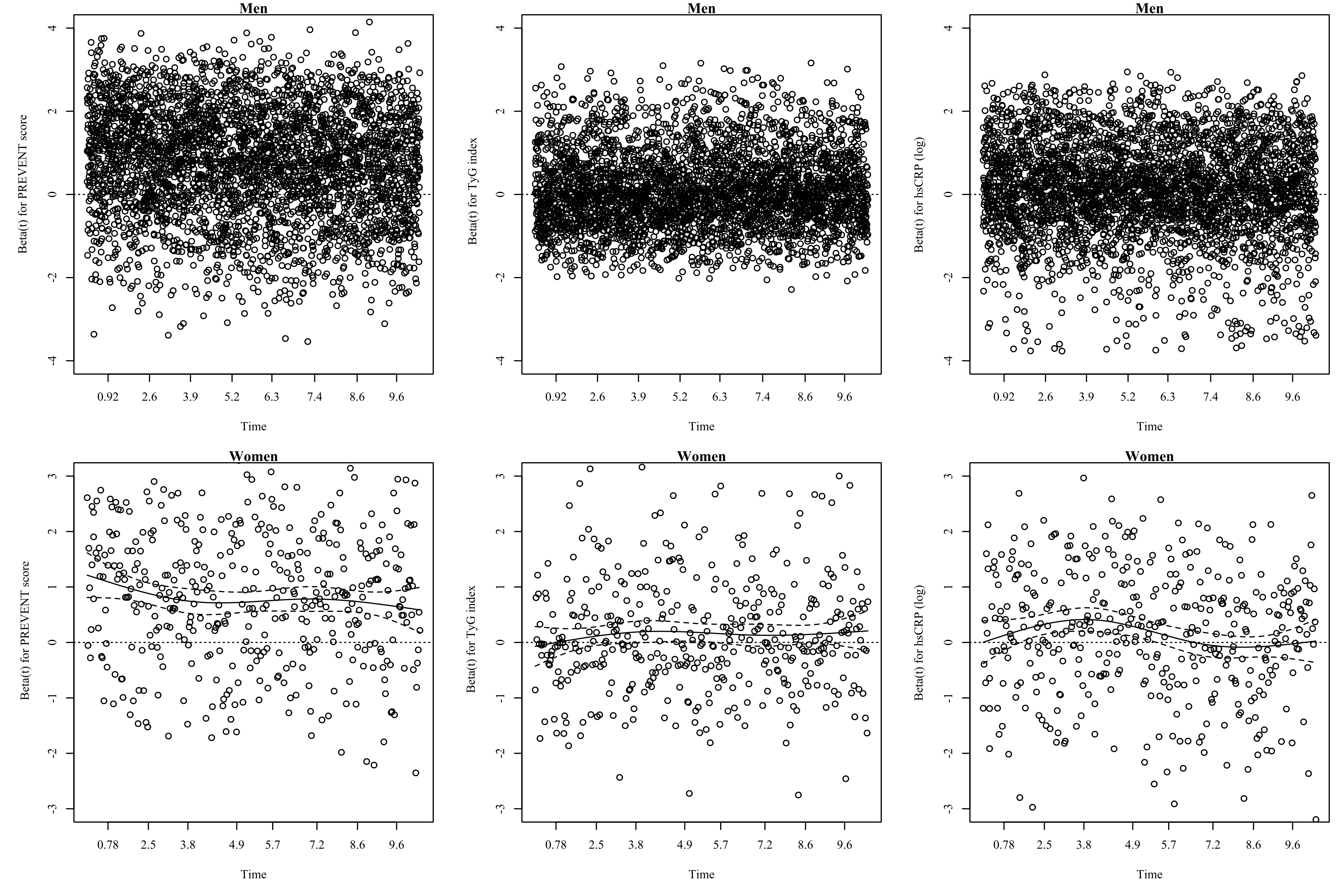


**Supplemental Figure 1. Schoenfeld Plots**

**(A)** **Men**


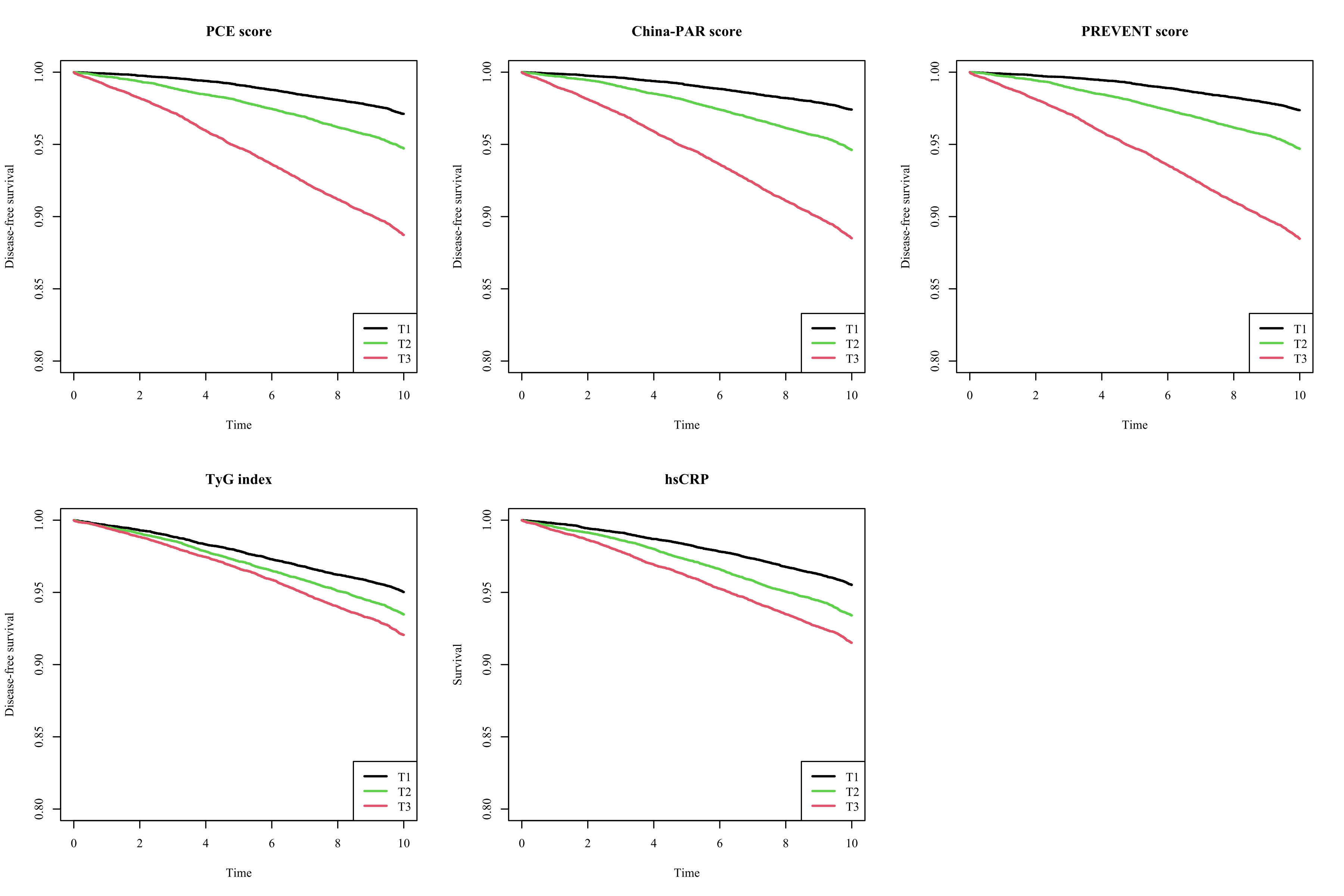


**(B)** **Women**


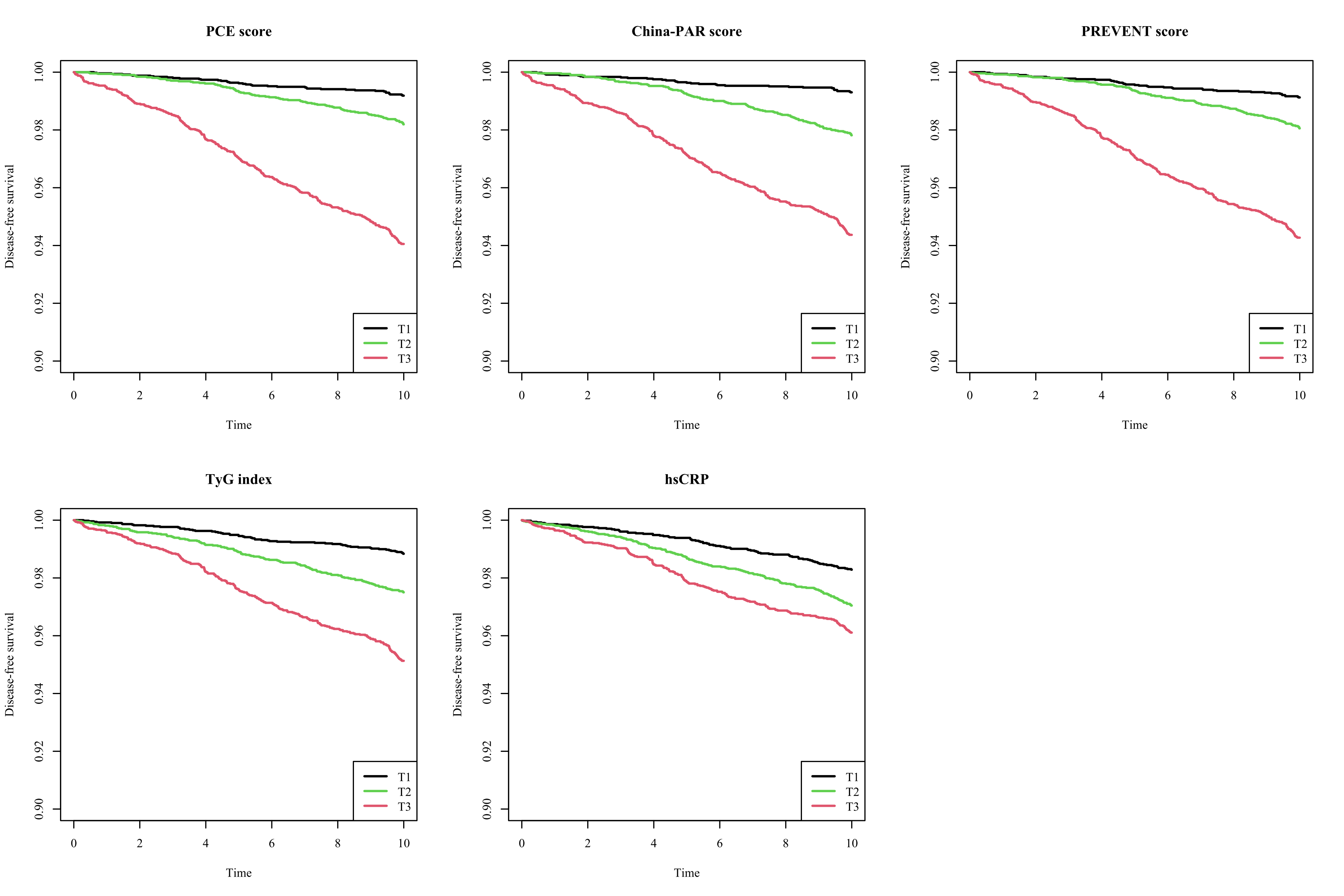


**Supplemental Figure 2. Kaplan-Meier Curve**

**(A) With multiple imputation**


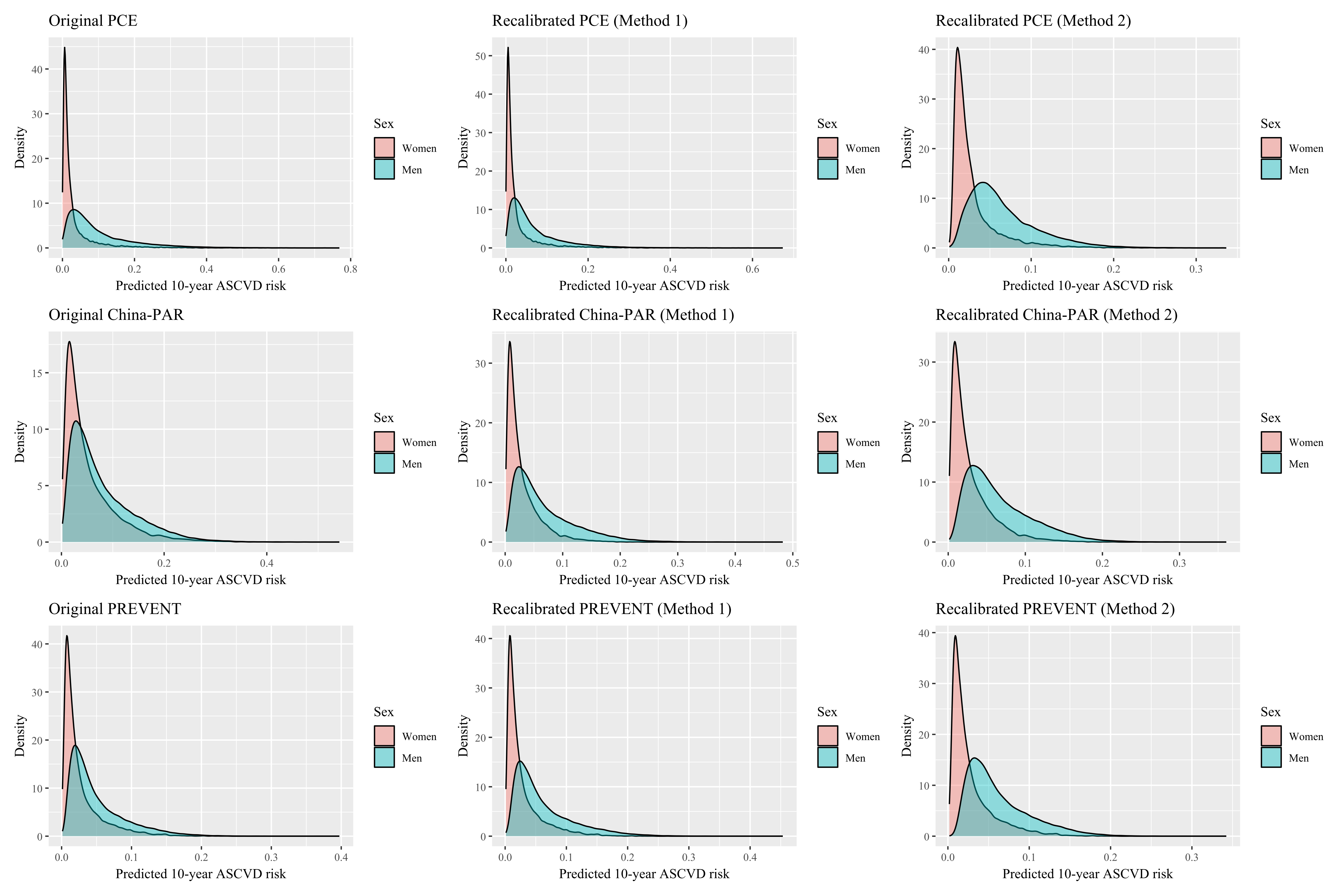


**(B) Complete-case analysis**


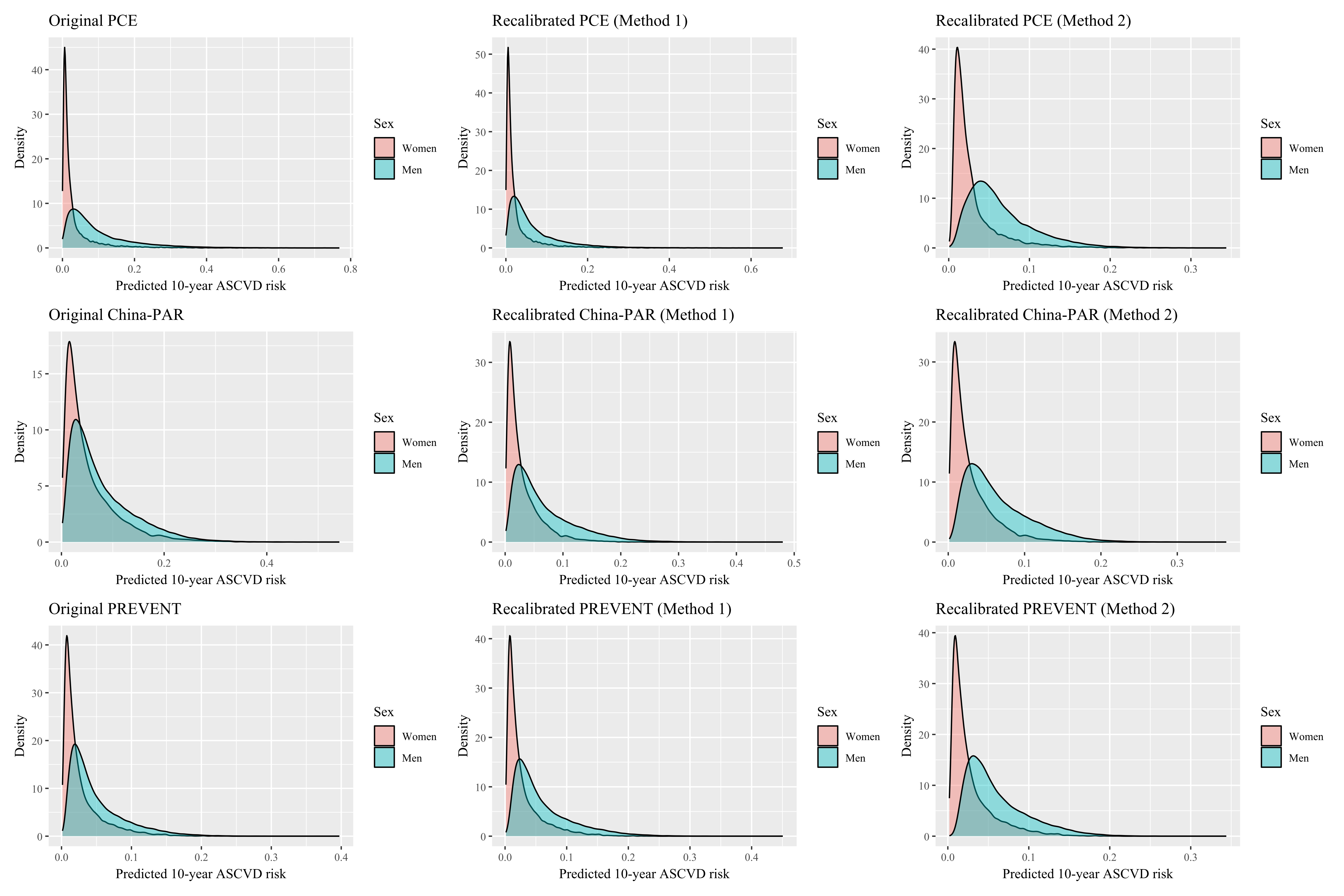


**Supplemental Figure 3. Density Plot of the** **10-Year Original and Recalibrated ASCVD Risk**
